# Dissecting the impact of molecular T-cell HLA mismatches in kidney transplant failure: A retrospective cohort study

**DOI:** 10.3389/fimmu.2022.1067075

**Published:** 2022-11-24

**Authors:** William Lemieux, David Fleischer, Archer Yi Yang, Matthias Niemann, Karim Oualkacha, William Klement, Lucie Richard, Constantin Polychronakos, Robert Liwski, Frans Claas, Howard M. Gebel, Paul A. Keown, Antoine Lewin, Ruth Sapir-Pichhadze

**Affiliations:** ^1^ Centre for Outcomes Research and Evaluation (CORE), Research Institute of McGill University Health Centre, Montréal, QC, Canada; ^2^ Medical Affairs & Innovation, Héma-Québec, Montréal, QC, Canada; ^3^ Department of Mathematics and Statistics, McGill University, Montreal, QC, Canada; ^4^ Research and Development, PIRCHE AG, Berlin, Germany; ^5^ Department of Mathematics, Université du Québec à Montreal, Montreal, QC, Canada; ^6^ Division of Organ Donation and Transplantation, Canadian Blood Services, Ottawa, ON, Canada; ^7^ Transfusion medicine/Reference Laboratory, Héma-Québec, Montréal, QC, Canada; ^8^ Department of Pediatrics, The Research Institute of the McGill University Health Centre and the Montreal Children’s Hospital, Montréal, QC, Canada; ^9^ Department of Pathology, Dalhousie University, Halifax, NS, Canada; ^10^ Department of Immunology, Leiden University Medical Centre, Leiden, Netherlands; ^11^ Pathology and Laboratory Medicine, Emory University, Atlanta, GA, United States; ^12^ Department of Medicine, University of British Columbia, Vancouver, BC, Canada; ^13^ Faculty of Medicine and Health Sciences, Université de Sherbrooke, Sherbrooke, QC, Canada; ^14^ Division of Nephrology and the Multi-Organ Transplant Program, Royal Victoria Hospital, McGill University Health Centre, Montréal, QC, Canada; ^15^ Department of Epidemiology, Biostatistics and Occupational Health, McGill University, Montréal, QC, Canada

**Keywords:** human leukocyte antigens, PIRCHE-II, death-censored graft failure, feature selection, network analysis

## Abstract

**Introduction:**

Kidney transplantation is the optimal treatment in end-stage kidney disease, but *de-novo* donor specific antibody development continues to negatively impact patients undergoing kidney transplantation. One of the recent advances in solid organ transplantation has been the definition of molecular mismatching between donors and recipients’ Human Leukocyte Antigens (HLA). While not fully integrated in standard clinical care, cumulative molecular mismatch at the level of eplets (EMM) as well as the PIRCHE-II score have shown promise in predicting transplant outcomes. In this manuscript, we sought to study whether certain T-cell molecular mismatches (TcEMM) were highly predictive of death-censored graft failure (DCGF).

**Methods:**

We studied a retrospective cohort of kidney donor:recipient pairs from the Scientific Registry of Transplant Recipients (2000-2015). Allele level HLA-A, B, C, DRB1 and DQB1 types were imputed from serologic types using the NMDP algorithm. TcEMMs were then estimated using the PIRCHE-II algorithm. Multivariable Accelerated Failure Time (AFT) models assessed the association between each TcEMM and DCGF. To discriminate between TcEMMs most predictive of DCGF, we fit multivariable Lasso penalized regression models. We identified co-expressed TcEMMs using weighted correlation network analysis (WGCNA). Finally, we conducted sensitivity analyses to address PIRCHE and IMGT/HLA version updates.

**Results:**

A total of 118,309 donor:recipient pairs meeting the eligibility criteria were studied. When applying the PIRCHE-II algorithm, we identified 1,935 distinct TcEMMs at the population level. A total of 218 of the observed TcEMM were independently associated with DCGF by AFT models. The Lasso penalized regression model with post selection inference identified a smaller subset of 86 TcEMMs (56 and 30 TcEMM derived from HLA Class I and II, respectively) to be highly predictive of DCGF. Of the observed TcEMM, 38.14% appeared as profiles of highly co-expressed TcEMMs. In addition, sensitivity analyses identified that the selected TcEMM were congruent across IMGT/HLA versions.

**Conclusion:**

In this study, we identified subsets of TcEMMs highly predictive of DCGF and profiles of co-expressed mismatches. Experimental verification of these TcEMMs determining immune responses and how they may interact with EMM as predictors of transplant outcomes would justify their consideration in organ allocation schemes and for modifying immunosuppression regimens.

## Introduction

Solid organ transplantation is the definitive treatment for eligible patients with end stage organ failure (1). Among all solid organs, kidney transplants are the most frequent. Yet, premature graft failure makes re-transplantation a common event. Of the various causes of premature kidney graft failure, rejection is the leading cause (2). Rejection involves the immunological recognition of the graft as foreign and its attack by the host’s immune system. While this process can be mitigated by induction and maintenance immunosuppression, poor adherence, or dose reduction render patients more vulnerable to developing immune injury because of incompatibility of human leukocyte antigens (HLA) with their donors.

Immune-related injury is less likely when HLA compatibility is greater between patients and donors. Yet, the fact that more than 32,000 HLA alleles have been identified makes complete HLA allelic matching of unrelated donors and patients exceedingly difficult (3). In recent decades, algorithms developed to study the degree of molecular HLA mismatch between donors and recipients have gained traction (4). HLAMatchmaker, for example, considers eplets, which represent polymorphic residues within a radius of 3.0-3.5 Å of a given sequence position on the molecular surface (5, 6). Eplet mismatches (EMM) were found to be predictive of immune injury and graft failure (7–10). Efforts to optimize compatibility at the level of HLA eplets offer the advantage of avoiding the incompatibilities that tend to inform antibody development. Also, the likelihood of identifying eplet compatible donors is increased by virtue of eplets being common to HLA alleles of the same locus and across loci (11, 12). While the literature primarily considered cumulative load of EMM as a predictor of transplant outcomes, EMM may differ in their association with inferior transplant outcomes (13), with individual high-risk eplet mismatches (and/or EMM co-expressed with them) informing graft failure risk with greater discrimination than the overall EMM load (14).

It is also important to recall that most EMM were envisioned to explain immune recognition by B-cells, which represents only part of the sequence of events leading to donor-specific antibody (DSA) development and allograft rejection. While the B-cell response is pivotal in DSA development, the T-cell response is also of importance (15). To address the indirect immune response by T-cells, a complementary approach was developed in the form of the Predicted Indirectly ReCognizable HLA Epitopes (PIRCHE-II) algorithm (16). The PIRCHE-II approach uses the protein sequence of HLA alleles to predict the allogeneic foreign peptides that can be presented within the recipients’ HLA Class II groove. Several reports suggest that the mismatch load of donor-derived peptides, which are absent in the recipient self-peptidome, but expressed by recipient HLA, are predictive of immune response and graft failure (17–19). As the peptides used for PIRCHE-II represent theoretical T-cell epitopes, it is important to verify their association with outcomes like graft failure. We posit that while the load of PIRCHE-II may be predictive of graft failure, individual PIRCHE-II peptides, or T-cell molecular mismatch (TcEMM), might carry different risks.

Like EMM, the analysis of PIRCHE-II is highly susceptible to the complexities of high dimensionality and relatedness between PIRCHE-II peptides. The relatedness (measured by correlation) is, at least in part, due to the tendency of HLA genes to be in linkage disequilibrium, and for PIRCHE-II peptides to be present in relation to multiple HLA molecules (20). Consequently, a large cohort is necessary to disentangle which of the mismatched PIRCHE-II peptides are most predictive of transplant outcomes. While both EMM and TcEMM may interact to inform transplant outcomes, one must first evaluate TcEMM as independent predictors. We thus conducted a retrospective cohort study to verify the independent association of mismatched PIRCHE-II peptides and death-censored graft failure (DCGF), assess the relatedness between mismatched peptides, and determine TcEMM most predictive of DCGF.

## Methods

### Data source

This study used data from the Scientific Registry of Transplant Recipients (SRTR). The SRTR data system includes data on all donors, wait-listed candidates, and transplant recipients in the US, submitted by the members of the Organ Procurement and Transplantation Network (OPTN). The Health Resources and Services Administration (HRSA), U.S. Department of Health and Human Services provides oversight to the activities of the OPTN and SRTR contractors. The McGill University Health Center research ethics board approved the study.

### Study design

We performed a retrospective cohort study in kidney transplant recipients (KTRs) transplanted from January 1^st^, 2000 to January 1^st^, 2015. We excluded KTR who received multi-organ transplants or who had prior transplants. We also excluded KTRs who exhibited preformed antibodies (based on peak panel reactive antibody (PRA) above 0% irrespective of the cause for sensitization, those missing PRA, and those who experienced primary graft non-function.

### HLA typing and PIRCHE-II assignment

**Figure 1:**
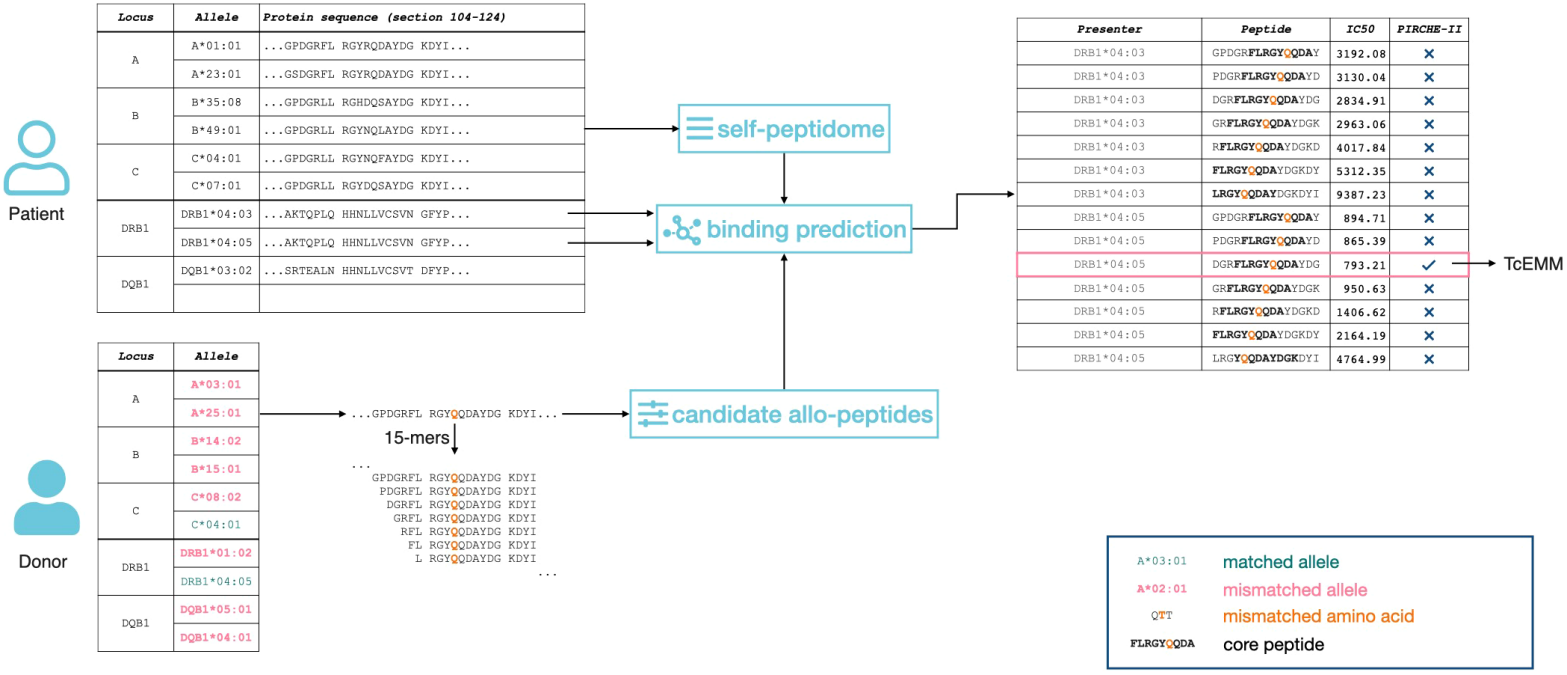
Representation of the PIRCHE-II algorithm. 15-amino acid long peptides (15-mers) are calculated for both recipient and donor. The subset of peptides differing between donor and recipient that are predicted to bind to the recipient`s Class II HLA are considered as TcEMMs.

Allele-level HLA-A, -B, -C, -DRB1, and -DQB1 genotypes were imputed using the National Marrow Donor Program algorithm as previously described (13). This corresponds to the best performing imputation strategy currently available with limited implications to the identity of Class I but more so for Class II PIRCHE-II and EMM (21, 22). The PIRCHE-II algorithm (version 3.2, IMGT/HLA version 3.46) was applied to allele-level genotypes and yielded mismatched peptides, representing theoretical TcEMMs, as well as the PIRCHE-II score (**Figure 1**). The peptides considered the subset of 15 amino acid frames from the donors’ HLA proteins capable of binding to the recipient’s HLA-DRB1. Of these, 9-amino-acid-long cores, absent from the recipients’ repertoire of cores, and directly binding to the recipients’ HLA-DRB1 were considered for TcEMM analysis. Each of the recipients’ HLA-DRB1 as well as TcEMM (determined for donor:recipient pairs) were modeled as binary variables (present/absent) yielding a TcEMM expression matrix.

### Outcome and covariates

The primary outcome was time to DCGF, defined as return to dialysis or re-transplantation. All baseline characteristics of recipients (age, sex, time on dialysis, insurance coverage, and HLA-DRB1), donors (age, sex, and type) and transplants (transplant era, induction agent, calcineurin inhibitor type, steroid use, cold-ischemia time, and donor-recipient weight ratio) were considered as potential covariates (see **Supplementary Table I**).

### Statistical analyses

Eligible KTRs were followed until graft-failure, death, or administratively censored on May 31^st^, 2015. We performed univariable and multivariable (considering all covariates) regression using an Accelerated Failure Time (AFT) model (under a Weibull distribution) of the log-transformed PIRCHE-II score. Missing values for covariates were previously imputed by Multiple Imputation by Chained Equations (MICE) (13). In accordance with previous studies with PIRCHE-II score, a value of 1 was added to the scores prior to the log-transformation (23). As we found that for some TcEMM, the proportionality of hazards assumption was violated in the models assessing risk of DCGF (**Supplementary Table 1**), instead of Cox proportional hazards (CoxPH) models, we fit the AFT models. We then compared the Akaike Information Criterion (AIC) scores of the models built under the CoxPH assumption and the AFT models to determine the best-fitting model for single TcEMM. For each TcEMM, the hazard ratio (HR) was calculated, with HRs above 1 indicating a higher risk in the presence of a TcEMM and HRs below 1 indicating a decreased risk. We used the Benjamini-Hochberg procedure to control the false discovery rate as we performed multiple tests (24). Finally, we reported on the frequency of each TcEMM in the population of donor-recipient pairs and compared the frequency of TcEMM associated with DCGF vs. not.

To identify a subset of TcEMMs highly predictive of DCGF, while considering highly co-expressed TcEMM, we fitted penalized Cox regression models. We used the Least Absolute Shrinkage and Selection Operator (Lasso) method for feature selection and regularization of data (25, 26). Lasso performs L1-norm regularisation on a Cox regression model. To select the appropriate regularisation parameter, 10-fold cross-validation was used and cross-validation error was minimized. TcEMM selected by Lasso, were then subject to post-selection inference. The latter procedure helped inform valid confidence intervals for the selected coefficients verifying that all variables included in the final model were relevant. To identify residue positions differing between selected and unselected TcEMM, we compared amino acid residues using the DiffLogo R package.

Lasso penalized regression identifies, from a group of potential variables, the most informative predictors in relation to an outcome of interest. In this process, one of several correlated variables could be selected as the important predictor. Yet, either the selected predictor, or the variables correlated with it, could be causally related to the outcome. To represent the interrelatedness between TcEMMs, we used Weighted Correlation Network Analysis (WGCNA) and computed the predictor’s correlation matrix. Using a correlation threshold of 0.74 (corresponding to an adjacency threshold of 0.3 (13)), we generated an adjacency matrix and provided a visual representation of subsets of highly correlated TcEMMs.

We performed a sensitivity analysis to evaluate how the output of the PIRCHE-II algorithm may vary when considering the most recent update to the IMGT/HLA version 3.47 (PIRCHE-II 3.3) in comparison to the prior version 3.46 (PIRCHE-II 3.2). We compared the mismatched TcEMMs generated by the PIRCHE-II algorithm and evaluated the impact of this change on the identity of TcEMM at the population and at the donor-recipient pair level. Statistical analyses were performed using several packages [WGCNA (27, 28), glmnet (25, 26), survival (29, 30), selectiveInference (31), and DiffLogo (32)] from version 4.1.2 R statistical computing software (33).

## Results

Upon application of the exclusion criteria, we identified 118,309 eligible KTR (see **Supplementary Figure 1**, **Supplementary Table 1** for the study flow diagram and baseline characteristics). The median follow-up for the analytic cohort was 6.39 years (Interquartile range (IQR) 3.12 - 10.01 years) and a total of 19,945 (~ 17%) DCGF events occurred within a median of 3.12 years (IQR 1.42 - 6.54) post-transplant. In this cohort, log-transformed PIRCHE-II scores were associated with an increased risk for DCGF, with significant HRs of 1.16 (95% Confidence Interval [95%CI] = 1.14-1.17) and 1.14 (95%CI = 1.12-1.15) for univariable and multivariable models, respectively.

### DCGF risk associated with single TcEMM

**Figure 2:**
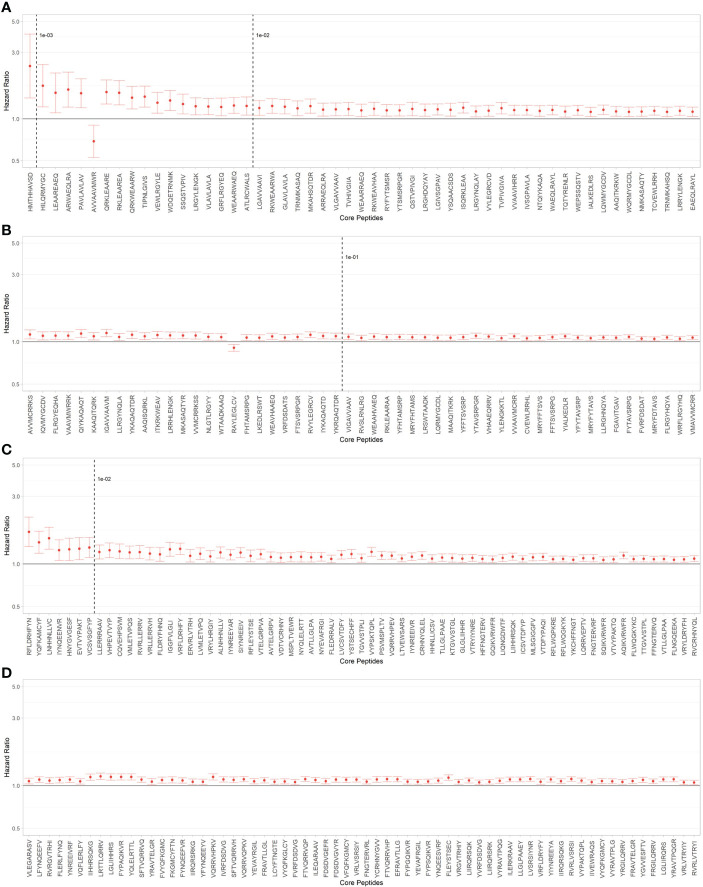
TcEMM associated with death-censored graft failure (DCGF). Hazard ratios and 95% confidence intervals (95%CI) for DCGF by TcEMM in Accelerated Failure Time (AFT) multivariable models adjusted for covariates included in Supporting Table 1 and the recipient’s HLA-DRB1. TcEMM originating from Class I **(A, B)** and Class II HLA **(C, D)** present in ≥50 recipients and meeting the threshold of statistical significance when correcting for multiple testing are presented by decreasing frequency. Dashed lines indicate logarithmic frequency bins.

Using the PIRCHE-II algorithm, we identified 1935 distinct TcEMM in our cohort, 1236 derived from HLA Class I and 699 from HLA Class II. Each TcEMM was present in a median of 1080 (IQR 46 - 4790; range 1 to 26,735) donor:recipient pairs. Of the possible multivariable AFT models considering each TcEMM as the main exposure, the Weibull distribution offered the best fit as measured by the AIC score, and all distributions were better than the fit from CoxPH models (**Supplementary Table 2**). **Figure 2** presents HRs of DCGF and 95% CI for TcEMM observed in ≥50 donor:recipient pairs and after correcting for multiple testing. The complete list of TcEMM and their associated risk estimates for DCGF is available in **Supplementary Table 3**.

### Frequencies of TcEMM in the cohort

When assessing whether the frequencies of TcEMM differed between those TcEMM that were predictive of DCGF and those that were not, we observed overlapping distributions (median (range): 6.27x10^-03^ (8.45x10^-06^ - 2.26x10^-01^) and 9.44x10^-03^ (8.45x10^-06^ - 2.02x10^-01^), respectively).

### Dissecting TcEMM most predictive of DCGF risk

**Figure 3:**
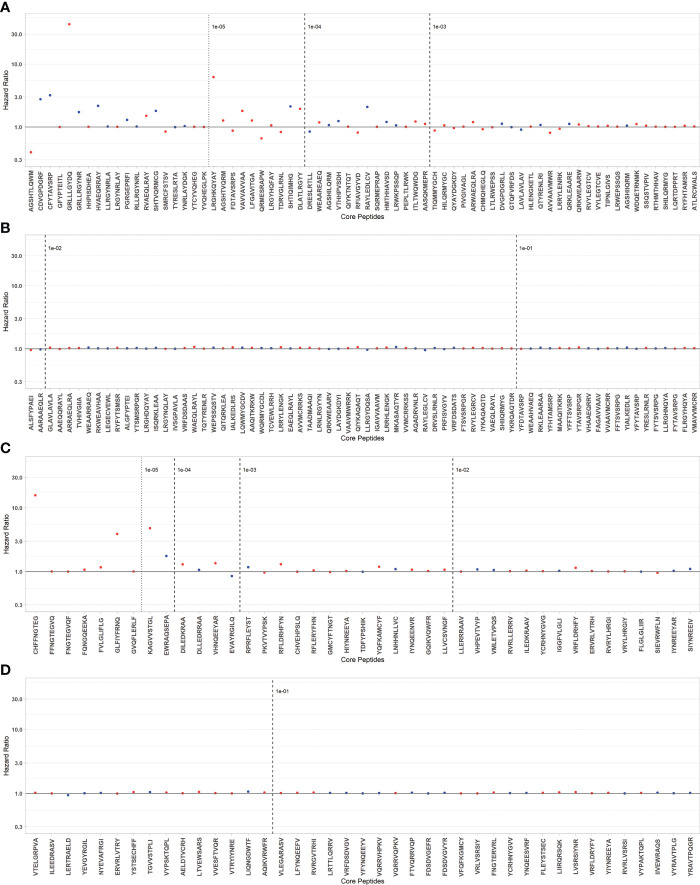
TcEMM selected as predictors of DCGF by Lasso penalized regression model. Hazard ratios for DCGF by selected TcEMMs originating from Class I **(A, B)** and Class II HLA **(C, D)** are presented by decreasing frequency. TcEMMs maintaining their status as statistically significant predictors following post-selection inference are represented in blue. Models were adjusted for the covariates included in Table 1 and the recipient’s HLA-DRB1. Dashed lines indicate logarithmic frequency bins with TcEMM to the left of the 10^-5^ frequency being observed only in a single individual from the cohort.

To fit a parsimonious model including the selection of TcEMM most predictive of DCGF, we fit regression models and applied a Lasso penalization term. A subset of 104 Class I and 82 Class II derived TcEMM were identified as predictors of DCGF (**Figure 3**, **Supplementary Table 4**). Of these, 56 Class I and 30 Class II derived TcEMM were significant by the post-selection inference test. When assessing whether the increased DCGF risk was informed by differences in amino acids at one or more positions of the TcEMM selected by the Lasso (and verified by post-selection inference) in comparison to those that were not selected, we did not find significant differences.

### Profiles of TcEMMs

**Figure 4:**
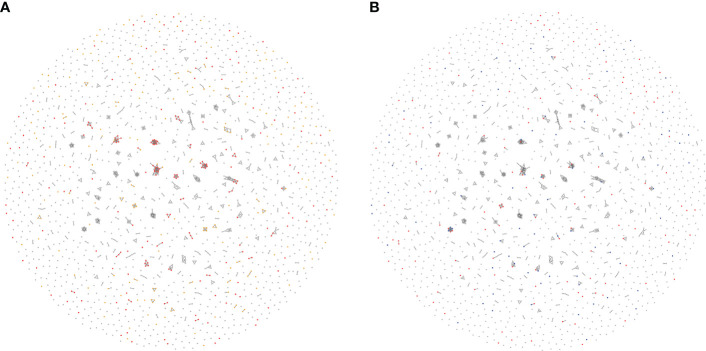
Interrelatedness of TcEMMs selected by Lasso penalized regression. Each *node* represents a TcEMM associated with death-censored graft failure (DCGF) in Accelerated Failure Time (AFT) Models **(A)** and Lasso penalised regression **(B)**. In panel **(A)**, TcEMMs associated with DCGF by AFT models when accounting for multiple testing present in ≥50 donor: recipient pairs and those in <50 donor:recipient pairs are represented in Red and Yellow, respectively. In panel **(B)**, TcEMMs selected by the penalized regression model and the subset of TcEMMs that remained significant following post-selection inference are represented in Red and Blue, respectively. In both Figures, only *edges* connecting between highly co-expressed TcEMM are shown. https://wlemieux03.github.io/TcEMM/ provides more detailed and interactive graph.

Co-expressed TcEMMs observed in the entire cohort when undergoing WGCNA are presented in **Figure 4A**. A total of 738 TcEMM appeared within 242 profiles, with profiles comprising of 2 to 18 TcEMMs. The remaining TcEMM appeared as singletons. Rather than the selected predictor TcEMM, a co-expressed TcEMM (represented in the profile) may in fact be causally related to increased DCGF risk. The relation between Lasso selected TcEMM and mismatched profiles is visualized in **Figure 4B** (see also https://wlemieux03.github.io/TcEMM/for interactive graphs).

### Sensitivity analyses

We conducted sensitivity analyses to address IMGT/HLA version updates. Realising the PIRCHE-II algorithm is likely to yield distinct TcEMM as the IMGT/HLA database is updated, we evaluated the differences between the TcEMM observed in the IMGT/HLA version the PIRCHE-II algorithm relied on for our main analyses (3.46) with those observed with the most recent version (3.47) (**Supplementary Table 5**). The updated version resulted in 144 different TcEMM affecting only 171 donor:recipient pairs of the analytic cohort (0.14%); among those, 84 rare TcEMM were no longer observed in the cohort, and 60 additional TcEMM were different in some donor:recipient pairs.

## Discussion

Using a data driven approach, we studied a retrospective cohort of KTR from the SRTR to identify selected TcEMM most predictive of DCGF. In contrast to the traditional TcEMM analysis, which yields PIRCHE-II scores (akin to TcEMM load), our analyses sought to distinguish between TcEMMs associated with an increased risk of DCGF and those that were not. AFT models followed by penalized regression models and post-selection inference informed an up to 20-fold reduction in the number of TcEMMs associated with DCGF. These findings pave the way to more targeted experimental verification of TcEMMs. Such experimental verification will then lend support to their consideration in decisions on organ allocation and personalized management of KTR.

There is a need for novel biomarkers informing risk for immune and non-immune-mediated outcomes in kidney transplantation (34, 35). With the wider acceptance of molecular mismatch (5), or EMM load, as a predictor of transplant outcomes, there has been a growing interest in determining how the T-cell counterpart may contribute to transplant outcomes. Prior publications proposed TcEMM score thresholds for risk stratification of kidney transplant recipients (17–19, 36, 37). To further refine the assessment of risk by TcEMM, we sought to study how the identity of TcEMM informs risk. Sufficient statistical power was needed to study high dimensional data and disentangle which of the interrelated TcEMMs represent the most important predictors. Recently, in a study of child-specific antibodies in pregnant women, Niemann and colleagues identified a subset of TcEMM proposed to be highly immunogenic (19). Challenged by a small sample size, none of the TcEMMs were found to significantly associate with DSA development. This is despite previously observed associations between TcEMM score and child-specific antibody development (19). Interestingly, of the 60 peptides highlighted in the analysis by Niemann and colleagues, 41 were also observed in our cohort, and 14 of these were selected by one or more of the analyses reported in this manuscript. However, it should be noted that 56 of the 60 TcEMM identified by Niemann and colleagues originated from or presented by HLA-DRB3/4/5, -DQA1, -DPB1, or -DPA1, which are unavailable in the analytic dataset. There are several methodological differences, including the strategy in TcEMM ascertainment, the context of the sensitizing event studied (pregnancy vs. transplantation), and outcomes studied (DSA vs. DCGF). Niemann et al. also identified that position 4 of the peptides was different between high and low-risk TcEMM, with serine and glutamic acid being more frequent in high-risk TcEMM and phenylalanine and aspartic acid more frequent in low-risk TcEMM. When using DiffLogo in our dataset, we were not able to observe significant differences at any amino-acid position for TcEMM selected by Lasso, to support the notion that enrichment of amino acid differences distinguishes high-risk TcEMM.

It has been proposed that the frequency of TcEMM among donor:recipient pairs may affect the likelihood of it being predictive of DCGF. Inevitably, rare albeit high risk TcEMM are likely to yield imprecise relative risk estimates (**Figure 2**). Importantly, the high-risk for DCGF observed with certain TcEMMs is more likely informed by the properties of molecular mismatches rather than the frequency with which they are observed. This impact of rare TcEMM emphasizes the need to study large and well specified prospective multicenter cohorts with unambiguous allele-level HLA types with information on longitudinal exposure to immunosuppression. When planning for future clinical applications, donor:recipient incompatibility due to frequently observed high-risk TcEMMs can be readily avoided as kidneys with high-risk cores could be allocated to candidates expressing similar cores. In the case of rare high risk TcEMM on the other hand, the likelihood of finding a compatible candidate is lower, and it may be important to plan for national organ sharing as a strategy to allocate these organs to HLA compatible candidates.

Our study is the first to demonstrate an association between PIRCHE-II and DCGF in SRTR as well as the first attempt to identify TcEMM most predictive of DCGF. Nevertheless, we would like to acknowledge several limitations of the analyses and outline future research directions. First, while leveraging the analytical power enabled by the large SRTR dataset, which includes diverse populations of KTR, compensating for the sparsity of TcEMMs, it is vital to acknowledge that the TcEMM estimation is dependent on IMGT/HLA version considered and in the case of our analytic cohort relies on imputation (38). Unavailability of HLA-DRB3/4/5, -DQA1, -DPA1, and -DPB1 could lead to under- or over-estimation of TcEMM. Also, the analysis is limited considering the recipients’ presenting HLA was restricted to the HLA-DRB1 locus. Despite this, it is important to recall that there is a recognized utility for imputation, especially in the context of solid organ transplantation (22). Until large datasets with complete HLA genotypes at the allele-level of both donors and recipients are made available, our analyses can be used to inform analytical strategies when managing sparse data and considering all TcEMM, including rare ones. As the identity of rare peptides may vary from one cohort to another, both internal and external validation may be challenging. The consideration of rare HLA alleles, and those for which the complete sequence is not yet known, introduces another level of uncertainty. For these alleles, the PIRCHE algorithms considers the closest fully sequenced allele to inform on TcEMMs. While most such alleles differ from the closest alleles in intronic or noncoding regions, there could be differences between the assumed and true TcEMMs. Second, interrelatedness makes identification of TcEMMs that are causally related to graft failure challenging. Approximately a third of the TcEMM (38.14%) observed in our cohort were represented within highly correlated profiles (**Figure 3**). Future efforts for experimental verification of TcEMM must consider not only the Lasso selected high risk TcEMM but also those represented in TcEMM profiles. Also, as there is currently no direct measure of response to TcEMM, confirming the immunogenicity of high-risk TcEMM remains elusive (39). In lieu of experimental verification, demonstration that high risk TcEMM (or co-expressed TcEMM) associate with biopsy proven rejection and graft loss in future prospective cohort studies and clinical trials, will lend support to their clinical relevance. Third, the PIRCHE-II algorithm predicts TcEMM based on likelihood of binding by HLA Class II. It is important to recall that the chain of processes between peptide production, selection, and presentation may also be at play but these are currently not well elucidated. Last, our understanding of the interplay between TcEMM and EMM as determinants of transplant outcomes is also in evolution. T-cell and B-cell molecular mismatches have been shown to associate with DSA and antibody-mediated rejection (ABMR) in a small cohort of pediatric heart transplant patients, as well as in adult kidney recipients (40, 41). The study showed that patients with high TcEMM and EMM loads were at greatest risk. This would be in line with the immunological perspective behind humoral responses, with both B- and T-cell responses being involved in antibody production and class-switch. Future studies should assess whether combinations of TcEMM and high-risk EMM may better inform suboptimal transplant outcomes (13).

In conclusion, we sought to assess the contribution of individual TcEMM as determinants of DCGF and were able to distinguish increasingly smaller numbers of TcEMM most predictive of this endpoint through various approaches. Further validation of these TcEMM as determinants of transplant outcomes in large independent cohorts with allele-level HLA genotypes is needed. Furthermore, investigation of the properties that may contribute to immune injury are also needed. While TcEMM is not ready for clinical application, our observations provide the foundation for future studies assessing whether these high risk TcEMM (or the TcEMM co-expressed with them) inform other hard clinical endpoints like biopsy proven rejection. Such verification would justify their avoidance during organ allocation or modifications of posttransplant management when mismatches cannot be avoided. Importantly, with a better differentiation of risk profiles of particular EMM and TcEMM, future research is needed to clarify how they interact and inform long-term transplant outcomes.

## Data availability statement

The data analyzed in this study was obtained from the SRTR. To learn about the applicable licenses/restrictions and submit requests to access these datasets, please consult: https://www.srtr.org/requesting-srtr-data/data-requests/.

## Ethics statement

The studies involving human participants were reviewed and approved by McGill University Health Center research ethics board. Written informed consent from the participants’ legal guardian/next of kin was not required to participate in this study in accordance with the national legislation and the institutional requirements.

## Author contributions

Study conception and design: WL, RS-P. Acquisition of data: RS-P. Analysis and interpretation of data: WL, DF, AY, and RS-P. Drafting of manuscript: WL and RS-P Critical revision: WL, AY, MN, KO, WK, LR, CP, RL, FC, HG, PK, AL, RS-P. All authors contributed to the article and approved the submitted version.

## Funding

This research was enabled thanks to support provided by Genome Canada Large Scale Applied Research Program Award “Precision Medicine CanPREVENT AMR” funded by Genome Quebec, Genome British Columbia, Genome Alberta, and Canadian Institutes of Health Research, Calcul Québec (https://www.calculquebec.ca) and Compute Canada (https://www.computecanada.ca) through a Resource Allocation for Research groups (RAC cna-921-ac), and a MITACS Accelerate grant. RS-P is supported by Fonds de recherche du Quebec—Santé chercheur boursier clinicien award (grant no. 254386).

## Acknowledgments

The authors thank James Hanley, Sahir Bhatnagar, and Jesse Islam for their input on the analyses conducted.

## Conflict of interest

MN is an employee of PIRCHE AG that runs the PIRCHE web-portal.

The remaining authors declare that the research was conducted in the absence of any commercial or financial relationships that could be construed as a potential conflict of interest.

## Publisher’s note

All claims expressed in this article are solely those of the authors and do not necessarily represent those of their affiliated organizations, or those of the publisher, the editors and the reviewers. Any product that may be evaluated in this article, or claim that may be made by its manufacturer, is not guaranteed or endorsed by the publisher.

## Author disclaimer

The data reported here have been supplied by the Hennepin Healthcare Research Institute (HHRI) as the contractor for the Scientific Registry of Transplant Recipients (SRTR). The interpretation and reporting of these data are the responsibility of the author(s) and in no way should be seen as an official policy of or interpretation by the SRTR or the U.S. Government.

## Abbreviations

ABMR, Antibody-Mediated Rejection; AFT, Accelerated Failure Time; AIC, Akaike Information Criterion; 95% CI, 95% Confidence interval; DCGF, Death-Censored Graft-Failure; DSA, Donor-Specific Antibodies; EMM, Eplet mismatch; HLA, Human Leucocyte Antigen; HR, Hazard ratio; KTR, Kidney Transplant Recipient; LB, Lower bound of the 95% CI; MICE, Multiple Imputation by Chained Equations; PRA, Panel Reactive Antibodies; PIRCHE-II, Predicted Indirectly Recognizable HLA Epitopes presented by HLA Class II; SRTR, Scientific Registry of Transplant Recipients; TcEMM, T-cell molecular mismatch; UB, Upper bound of the 95% CI.
