# Supplementary figures and images for: Dissecting the impact of molecular T-cell HLA mismatches in kidney transplant failure: A retrospective cohort study
Source: Front Immunol. 2022 Nov 24;13:1067075. doi: 10.3389/fimmu.2022.1067075 (PMC9730505; doi:10.3389/fimmu.2022.1067075)

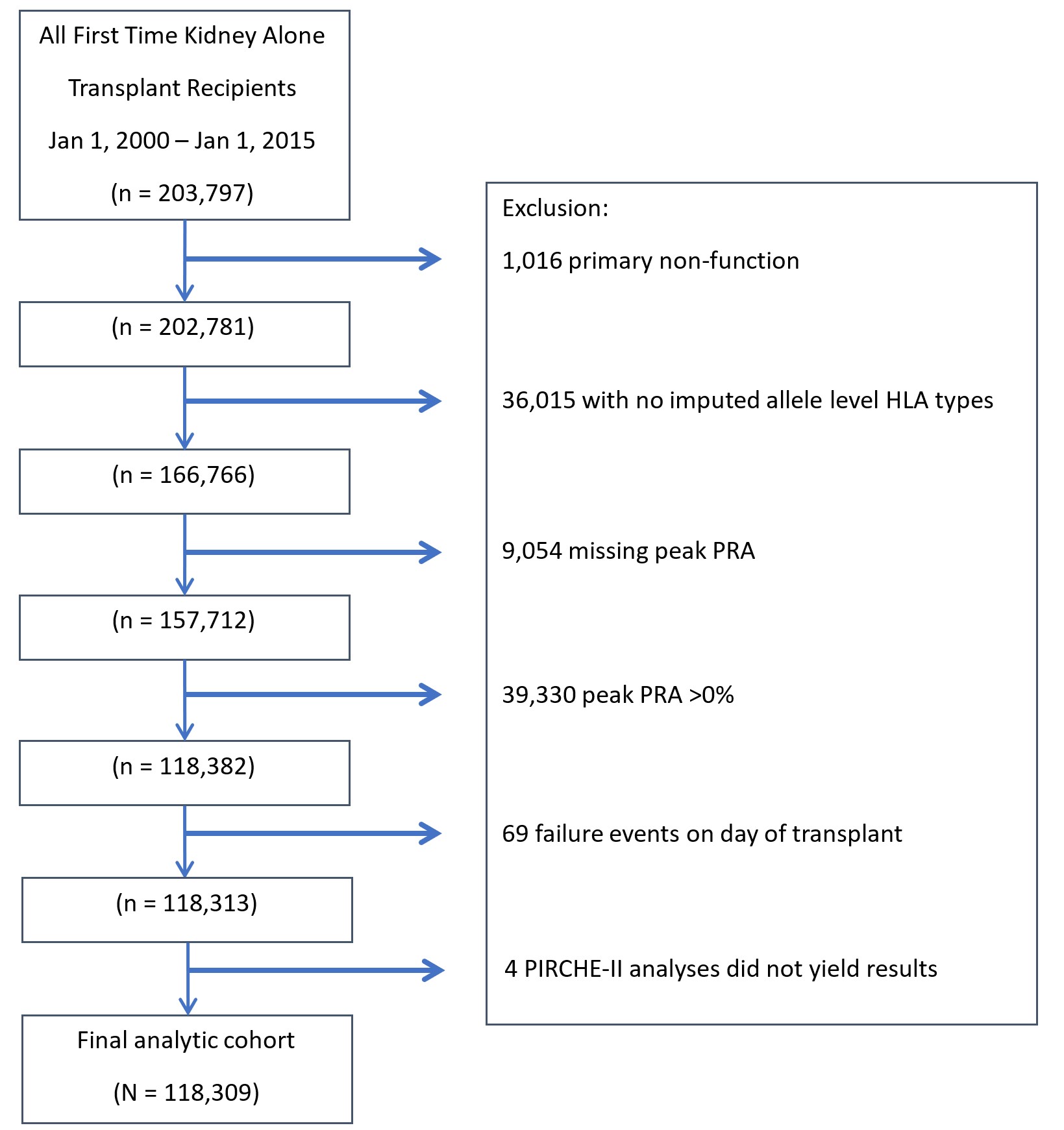

Supplement: Supplementary file 1 [file Image_1.jpg]
