# Supplementary material for: Dissecting the impact of molecular T-cell HLA mismatches in kidney transplant failure: A retrospective cohort study
Source: Front Immunol. 2022 Nov 24;13:1067075. doi: 10.3389/fimmu.2022.1067075 (PMC9730505; doi:10.3389/fimmu.2022.1067075)
Supplement: Supplementary file 2 [file Table_1.docx]

**Supplementary Table 1. Baseline characteristics of the donor:recipient pairs in the final analytical cohort.**

| Variable |  | n | % |
| --- | --- | --- | --- |
|  | **Recipients** |  |  |
| Age at transplantation | ≤14 | 4177 | 3.53 |
|  | 15-24 | 6490 | 5.49 |
|  | 25-44 | 30478 | 25.76 |
|  | 45-64 | 55395 | 46.82 |
|  | ≥65 | 21769 | 18.40 |
| Sex | Male | 78157 | 66.06 |
|  | Female | 40152 | 33.94 |
| Time on dialysis *(Mean, SD)* | *Months* | *36.86* | *32.59* |
|  | Missing | 23288 | 19.68 |
| Insurance type | Private | 49437 | 41.79 |
|  | Public | 68596 | 57.98 |
|  | None | 269 | 0.23 |
|  | Missing | 7 | 0.01 |
| Primary disease | Congenital | 16534 | 13.98 |
|  | Diabetes | 30086 | 25.43 |
|  | Glomerulonephritis | 30992 | 26.20 |
|  | Hypertension nephritis | 26893 | 22.73 |
|  | Other | 13692 | 11.57 |
|  | Missing | 112 | 0.09 |
|  | **Donors** |  |  |
| Age | ≤35 | 46418 | 38.91 |
|  | 36-45 | 27418 | 22.98 |
|  | 46-55 | 27804 | 23.30 |
|  | ≥56 | 16669 | 13.97 |
| Sex | Male | 61263 | 51.35 |
|  | Female | 57046 | 47.81 |
| Type | Deceased (SC) | 56919 | 47.71 |
|  | Deceased (EC) | 12749 | 10.69 |
|  | Living | 48641 | 40.77 |
|  | **Transplants** |  |  |
| Donor-recipient weight ratio | <0.9 | 45651 | 38.59 |
|  | 0.9-1.0 | 14379 | 12.15 |
|  | >1.0 | 50742 | 42.89 |
|  | Missing | 7537 | 6.37 |
| Induction agent | Campath | 9338 | 7.89 |
|  | IL2 Receptor blocker | 35189 | 29.74 |
|  | Thymoglobulin | 41348 | 34.95 |
|  | Other | 2435 | 2.06 |
|  | None | 29999 | 25.36 |
| Calcineurin inhibitor | Cyclosporine | 22242 | 18.80 |
|  | Tacrolimus | 87920 | 74.31 |
|  | None | 8147 | 6.89 |
| Steroid | Yes | 110342 | 93.27 |
|  | No | 7967 | 6.73 |
| Transplant era | 2000-2004 | 46060 | 38.93 |
|  | 2005-2009 | 42582 | 35.99 |
|  | 2010-2014 | 29667 | 25.08 |
| Cold ischemia time *(Mean, SD)* | *Hours* | *12.58* | *11.09* |
|  | Missing | 20236 | 17.10 |
