# Supplementary material for: Dissecting the impact of molecular T-cell HLA mismatches in kidney transplant failure: A retrospective cohort study
Source: Front Immunol. 2022 Nov 24;13:1067075. doi: 10.3389/fimmu.2022.1067075 (PMC9730505; doi:10.3389/fimmu.2022.1067075)
Supplement: Supplementary file 3 [file Table_2.docx]

**Supplementary Table 2. Example of Akaike Information Criterion (AIC) calculated for the multivariable Cox regression models and Accelerated Failure Time (AFT) models for the AASQKMEPR TcEMM**

| Distribution | Covariates+ Recipient HLA-DRB1  +AASQKMEPR | Covariates + Recipient HLA-DRB1 |
| --- | --- | --- |
| Cox PH | 439737.2 | 439740.9 |
| Exponential | 180902.8 | 180906.8 |
| Gaussian | 197926.3 | 197931.1 |
| Logistic | 200675.1 | 200679.5 |
| Log-logistic | 178538.4 | 178542.5 |
| Log-normal | 180441.8 | 180446.3 |
| Weibull | 178388.0 | 178391.5 |
