# Supplementary material for: Dissecting the impact of molecular T-cell HLA mismatches in kidney transplant failure: A retrospective cohort study
Source: Front Immunol. 2022 Nov 24;13:1067075. doi: 10.3389/fimmu.2022.1067075 (PMC9730505; doi:10.3389/fimmu.2022.1067075)
Supplement: Supplementary file 4 [file Table_3.docx]

**Supplementary Table 3. TcEMMs significantly associated with death-censored graft failure in multivariable accelerated failure time models**

| TcEMM | HLA Class | LB | HR | UB | p Value | HF^a^ | Highly-correlated coexpressed TcEMMs^b^ |
| --- | --- | --- | --- | --- | --- | --- | --- |
| AAQISQRKL | I | 1.033 | 1.094 | 1.158 | 0.012 | * | QRKLEAARV |
| AAQITKRKW | I | 1.040 | 1.129 | 1.226 | 0.020 | * |  |
| AAREAEQLR | I | 1.232e-06 | 1.467e-06 | 1.748e-06 | 0 |  |  |
| AASQRMEPR | I | 3.521e-06 | 4.138e-06 | 4.864e-06 | 0 |  | ASQRMEPRA* |
| AGSHTLQWM | I | 3.392e-06 | 3.989e-06 | 4.690e-06 | 0 |  |  |
| AHVAEQWRV | I | 1.978e-06 | 2.342e-06 | 2.773e-06 | 0 |  |  |
| ALNHHNLLV | II | 1.100 | 1.204 | 1.318 | 4.418e-04 | * | HNLLVCSVT |
| AQIKVRWFR | II | 1.087 | 1.147 | 1.212 | 6.972e-06 | * | LRTTLQRRV*, FYPAQIKVR*, YQLELRTTL*, LGLIIHHRS*, LIIHHRSQK*, IIHHRSQKG*, VTDFYPAQI* |
| ARFDSDVEV | II | 6.627e-06 | 7.726e-06 | 9.006e-06 | 0 |  |  |
| ARRAEQLRA | I | 1.044 | 1.162 | 1.293 | 0.029 | * | RAEQLRAYL, QRKWEAARR, WEAARRAEQ* |
| ARSESAQSK | II | 3.068e-05 | 3.506e-05 | 4.008e-05 | 0 |  |  |
| ARVAEQLRT | I | 1.224e-05 | 1.416e-05 | 1.638e-05 | 0 |  |  |
| ARVAEQRRT | I | 9.767e-06 | 1.133e-05 | 1.314e-05 | 0 |  |  |
| ARWAEQLRA | I | 1.210 | 1.619 | 2.165 | 0.007 | * |  |
| ASQRMEPRA | I | 3.521e-06 | 4.138e-06 | 4.864e-06 | 0 |  | AASQRMEPR* |
| ATLRCWALS | I | 1.062 | 1.235 | 1.435 | 0.029 | * |  |
| AVTELGRPV | II | 1.032 | 1.120 | 1.214 | 0.030 | * |  |
| AVTLLGLPA | II | 1.031 | 1.113 | 1.202 | 0.030 | * |  |
| AVVAAVMWR | I | 0.527 | 0.688 | 0.899 | 0.029 | * |  |
| AVVMCRRKS | I | 1.047 | 1.126 | 1.212 | 0.008 | * |  |
| AYLEGGCVE | I | 4.151e-05 | 4.727e-05 | 5.381e-05 | 0 |  | RAYLEGGCV* |
| CDVGPDGRF | I | 8.500e+05 | 1.018e+06 | 1.219e+06 | 0 |  | HHPISDHEA*, HMTHHPISD*, PISDHEATL* |
| CFSTSVSRP | I | 1.953e-06 | 2.313e-06 | 2.739e-06 | 0 |  |  |
| CHFFNGTEG | II | 9.089 | 64.770 | 461.566 | 2.481e-04 |  | FFNGTEGVQ*, FNGTEGVQF*, GVQFLERLF* |
| CQVEHPSVM | II | 1.090 | 1.227 | 1.381 | 0.004 | * | VHPEVTVYP* |
| CRHNYQLEL | II | 1.077 | 1.146 | 1.219 | 1.356e-04 | * |  |
| CRRKSSGGK | I | 1.083e-04 | 1.218e-04 | 1.370e-04 | 0 |  |  |
| CVDWLRRYL | I | 9.489e-05 | 1.069e-04 | 1.204e-04 | 0 |  | GTCVDWLRR* |
| CVEWLRRHL | I | 1.015 | 1.056 | 1.098 | 0.031 | * |  |
| DFLEQARAA | II | 2.298e-04 | 2.559e-04 | 2.850e-04 | 0 |  |  |
| DFVLQFKAM | II | 3.703e+05 | 4.386e+05 | 5.194e+05 | 0 |  |  |
| DFYPSQIKV | II | 1.288e-05 | 1.488e-05 | 1.720e-05 | 0 |  |  |
| DGRFLHGYH | I | 9.036e-05 | 1.019e-04 | 1.148e-04 | 0 |  | FLHGYHQYA* |
| DGRLLRGHN | I | 2.736e-05 | 3.132e-05 | 3.585e-05 | 0 |  |  |
| DLATLRGYY | I | 1.346 | 3.244 | 7.814 | 0.039 |  |  |
| DLLEDRRAL | II | 5.571e-06 | 6.509e-06 | 7.605e-06 | 0 |  |  |
| DLLEQARAA | II | 1.162e-05 | 1.345e-05 | 1.556e-05 | 0 |  | LLEQARAAV* |
| DLQTRNVKA | I | 2.067e-05 | 2.375e-05 | 2.728e-05 | 0 |  |  |
| DRESLRTLL | I | 1.443e-06 | 1.715e-06 | 2.039e-06 | 0 |  |  |
| DRNTQIYKT | I | 3.398e-05 | 3.878e-05 | 4.427e-05 | 0 |  |  |
| DRSHTLQRM | I | 1.785e-05 | 2.055e-05 | 2.365e-05 | 0 |  |  |
| EAEQLRAYL | I | 1.039 | 1.122 | 1.212 | 0.017 | * |  |
| EFRAVTLLG | II | 1.066 | 1.110 | 1.157 | 5.344e-06 | * | LLGLPAAEY*, RVRLVSRSI*, LVSRSIYNR*, ILERKRAAV*, IVRFDSDVG* |
| ERVRLVTRH | II | 1.030 | 1.141 | 1.263 | 0.049 |  |  |
| EVIVYPAKT | II | 1.736e-05 | 1.999e-05 | 2.302e-05 | 0 |  |  |
| EVRWFRNDQ | II | 2.117e-04 | 2.361e-04 | 2.632e-04 | 0 |  |  |
| EVRWFWNGQ | II | 1.111e-04 | 1.249e-04 | 1.404e-04 | 0 |  | SIEVRWFWN*, WFWNGQEEK* |
| EVTVYPAKT | II | 1.071 | 1.281 | 1.532 | 0.031 | * | HNYGVGESF* |
| EWSARSESA | II | 8.908e-06 | 1.034e-05 | 1.201e-05 | 0 |  |  |
| FDSDVGEFR | II | 1.030 | 1.076 | 1.125 | 0.007 | * |  |
| FDSDVGVYR | II | 1.057 | 1.104 | 1.154 | 8.978e-05 | * |  |
| FDTAVSRPS | I | 5.552e-06 | 6.487e-06 | 7.579e-06 | 0 |  |  |
| FFNGTEGVQ | II | 9.089 | 64.770 | 461.566 | 2.481e-04 |  | CHFFNGTEG*, FNGTEGVQF*, GVQFLERLF* |
| FFNGTERVQ | II | 1.029 | 1.080 | 1.134 | 0.011 | * | FYNQEEFVR*, FLERLFYNQ*, FLWQGKYKC*, YKCHFFNGT* |
| FFTSVSRPG | I | 1.037 | 1.076 | 1.117 | 8.381e-04 | * | MRYFFTSVS*, YFFTSVSRP* |
| FGAVITGAV | I | 1.029 | 1.066 | 1.104 | 0.002 | * | WRFLRGYHQ* |
| FGCDVGSDG | I | 9.761e-06 | 1.132e-05 | 1.313e-05 | 0 |  |  |
| FHNQEENVR | II | 1.308e-05 | 1.512e-05 | 1.747e-05 | 0 |  |  |
| FHTAMSRPG | I | 1.019 | 1.070 | 1.123 | 0.031 | * | MRYFHTAMS*, YFHTAMSRP* |
| FKGMCYFTN | II | 1.044 | 1.098 | 1.155 | 0.002 | * |  |
| FLDRYFHNQ | II | 1.045 | 1.167 | 1.304 | 0.030 | * |  |
| FLEDRRALV | II | 1.020 | 1.085 | 1.154 | 0.043 |  | LTVEWSARS* |
| FLEQARAAV | II | 6.755e-06 | 7.873e-06 | 9.175e-06 | 0 |  |  |
| FLERLFYNQ | II | 1.045 | 1.095 | 1.147 | 9.388e-04 | * | FLWQGKYKC*, YKCHFFNGT*, FYNQEEFVR*, RFLWQGKYK*, VQFLERLFY*, FFNGTERVQ* |
| FLESLFYNQ | II | 8.820e-05 | 9.945e-05 | 1.121e-04 | 0 |  | VQFLESLFY* |
| FLEYSTSEC | II | 1.089 | 1.140 | 1.193 | 1.430e-07 | * |  |
| FLGLGLIIH | II | 2.704 | 43.319 | 694.052 | 0.035 |  |  |
| FLHGYHQYA | I | 9.036e-05 | 1.019e-04 | 1.148e-04 | 0 |  | DGRFLHGYH* |
| FLNGQEEKA | II | 1.016 | 1.068 | 1.123 | 0.041 |  | YFYNQEESV |
| FLRGYEQHA | I | 1.037 | 1.105 | 1.176 | 0.011 | * |  |
| FLRGYHQSA | I | 3.430e-05 | 3.915e-05 | 4.468e-05 | 0 |  |  |
| FLRGYHQYA | I | 1.037 | 1.073 | 1.110 | 4.027e-04 | * |  |
| FLRGYRQHA | I | 3.707e-05 | 4.227e-05 | 4.820e-05 | 0 |  | LRGYRQHAY* |
| FLWQGKYKC | II | 1.031 | 1.082 | 1.137 | 0.009 | * | YKCHFFNGT*, FLERLFYNQ*, RFLWQGKYK*, FYNQEEFVR*, VQFLERLFY*, FFNGTERVQ* |
| FMLGLLFLG | II | 1.906e-05 | 2.192e-05 | 2.520e-05 | 0 |  | GFMLGLLFL*, VGGFMLGLL* |
| FNGTEGVQF | II | 9.089 | 64.770 | 461.566 | 2.481e-04 |  | CHFFNGTEG*, FFNGTEGVQ*, GVQFLERLF* |
| FNGTERVRF | II | 1.033 | 1.096 | 1.162 | 0.012 | * |  |
| FNGTERVRL | II | 1.026 | 1.068 | 1.112 | 0.007 | * | VRLLERCIY |
| FRAVTELGR | II | 1.033 | 1.077 | 1.123 | 0.003 | * |  |
| FRAVTLLGL | II | 1.044 | 1.090 | 1.138 | 6.129e-04 | * | LVSRSIYNR*, ILERKRAAV*, LLGLPAAEY*, RVRLVSRSI* |
| FRGILQRRV | II | 1.036 | 1.074 | 1.114 | 9.283e-04 | * | YEVAFRGIL*, FYPGQIKVR*, IIRQRSQKG*, CRHNYEVAF |
| FRKGQEEKT | II | 8.875e-05 | 1.001e-04 | 1.128e-04 | 0 |  | WFRKGQEEK* |
| FRNQKGHAG | II | 5.363e-06 | 6.269e-06 | 7.328e-06 | 0 |  |  |
| FTSVSRPGR | I | 1.029 | 1.080 | 1.133 | 0.010 | * |  |
| FTVQRRVHP | II | 1.066 | 1.113 | 1.162 | 1.063e-05 | * |  |
| FTVQRRVQP | II | 1.068 | 1.113 | 1.159 | 3.381e-06 | * | SFTVQRRVQ*, VQRRVQPKV*, RFLWQPKRE* |
| FVRFDSDAT | I | 1.015 | 1.051 | 1.088 | 0.025 | * |  |
| FVRFDSDVG | II | 1.016 | 1.059 | 1.104 | 0.032 | * | FYNQEEFVR* |
| FVYQFKGMC | II | 1.043 | 1.095 | 1.151 | 0.002 | * | VYQFKGMCY* |
| FWAVTELGR | II | 6.191e-05 | 7.013e-05 | 7.943e-05 | 0 |  |  |
| FYNQEEFVR | II | 1.043 | 1.089 | 1.136 | 7.578e-04 | * | VQFLERLFY*, FLERLFYNQ*, FFNGTERVQ*, FLWQGKYKC*, LFYNQEEFV*, YKCHFFNGT*, RFLWQGKYK*, FVRFDSDVG* |
| FYPAQIKVR | II | 1.095 | 1.153 | 1.213 | 5.700e-07 | * | YQLELRTTL*, LGLIIHHRS*, LRTTLQRRV*, IIHHRSQKG*, AQIKVRWFR*, VTDFYPAQI*, MLSGIGGFV*, LIIHHRSQK*, LSGIGGFVL, GLGLIIHHR* |
| FYPGQIKVR | II | 1.024 | 1.067 | 1.111 | 0.011 | * | YEVAFRGIL*, FRGILQRRV*, CRHNYEVAF |
| FYPGSIELR | II | 3.179e-05 | 3.632e-05 | 4.150e-05 | 0 |  |  |
| FYPSQIKVR | II | 1.029 | 1.070 | 1.112 | 0.004 | * | LIIRQRSRK*, VRGVTRHIY*, YEVAYRGIL*, SQIKVRWFR*, LCYFTNGTE*, VYQFKGLCY* |
| FYTAVSRPG | I | 1.044 | 1.081 | 1.119 | 1.046e-04 | * | YFYTAVSRP*, MRYFYTAVS* |
| GECVDGLRR | I | 1.262e-05 | 1.459e-05 | 1.687e-05 | 0 |  |  |
| GEITLTWQR | I | 1.011e-04 | 1.138e-04 | 1.280e-04 | 0 |  |  |
| GFMLGLLFL | II | 1.906e-05 | 2.192e-05 | 2.520e-05 | 0 |  | FMLGLLFLG*, VGGFMLGLL* |
| GFVLGLIFL | II | 5.243e-05 | 5.951e-05 | 6.755e-05 | 0 |  |  |
| GFYPAEITL | I | 0.108 | 0.111 | 0.115 | 0 |  |  |
| GFYPTEITL | I | 1.244e-05 | 1.438e-05 | 1.663e-05 | 0 |  |  |
| GGFMLGLLF | II | 8.682e-06 | 1.009e-05 | 1.172e-05 | 0 |  |  |
| GLAVLAVLA | I | 1.082 | 1.221 | 1.379 | 0.007 | * |  |
| GLGLIIHHR | II | 1.024 | 1.090 | 1.159 | 0.031 | * | LGLIIHHRS*, FYPAQIKVR*, YQLELRTTL*, LRTTLQRRV* |
| GMVSTGLVQ | II | 2.746e-05 | 3.143e-05 | 3.598e-05 | 0 |  | LVQNGDWTF*, YFYNQEENV* |
| GQIKVRWFR | II | 1.020 | 1.078 | 1.139 | 0.035 |  |  |
| GRFLRGYEQ | I | 1.051 | 1.215 | 1.405 | 0.038 |  |  |
| GRLLLGYDQ | I | 26.764 | 190.796 | 1.360e+03 | 1.525e-06 |  |  |
| GSHIIQSMY | I | 3.508e-05 | 4.002e-05 | 4.567e-05 | 0 |  |  |
| GSIELRWFR | II | 1.796e-05 | 2.067e-05 | 2.379e-05 | 0 |  | LRWFRNGQE* |
| GTCVDWLRR | I | 9.489e-05 | 1.069e-04 | 1.204e-04 | 0 |  | CVDWLRRYL* |
| GTGLFIYFR | II | 6.447e-06 | 7.518e-06 | 8.768e-06 | 0 |  | LLFLGTGLF*, VQRRVQPRV |
| GVQFLERLF | II | 9.089 | 64.770 | 461.566 | 2.481e-04 |  | CHFFNGTEG*, FFNGTEGVQ*, FNGTEGVQF* |
| HAGLQPTGF | II | 1.612e-05 | 1.858e-05 | 2.141e-05 | 0 |  | VYPSKSQPL* |
| HFFNGTERV | II | 1.024 | 1.084 | 1.148 | 0.027 | * |  |
| HHNLLICSV | II | 1.027 | 1.091 | 1.159 | 0.024 | * |  |
| HHPISDHEA | I | 8.500e+05 | 1.018e+06 | 1.219e+06 | 0 |  | CDVGPDGRF*, HMTHHPISD*, PISDHEATL* |
| HHSLLVCSV | II | 8.519e-06 | 9.899e-06 | 1.150e-05 | 0 |  | PLQHHSLLV* |
| HILQRMYGC | I | 1.218 | 1.727 | 2.449 | 0.012 | * |  |
| HMTHHAVSD | I | 1.408 | 2.390 | 4.054 | 0.007 | * |  |
| HMTHHPISD | I | 8.500e+05 | 1.018e+06 | 1.219e+06 | 0 |  | CDVGPDGRF*, HHPISDHEA*, PISDHEATL* |
| HNYGVGESF | II | 1.057 | 1.267 | 1.518 | 0.045 |  | EVTVYPAKT* |
| HPSLQSPIT | II | 2.940e-05 | 3.363e-05 | 3.845e-05 | 0 |  |  |
| HPSVTSPLA | II | 7.957e-05 | 8.984e-05 | 1.014e-04 | 0 |  |  |
| HSGLQPTGF | II | 0.489 | 0.494 | 0.498 | 0 |  |  |
| HVAEQWRVY | I | 8.862e-06 | 1.029e-05 | 1.195e-05 | 0 |  |  |
| IALKEDLRS | I | 1.037 | 1.120 | 1.210 | 0.021 | * |  |
| ICSVTDFYP | II | 1.028 | 1.084 | 1.142 | 0.015 | * | VTDFYPSQI, LCYFTNGTE*, VYQFKGLCY* |
| IGAVVAAVM | I | 1.086 | 1.152 | 1.223 | 2.901e-05 | * |  |
| IGGFVLGLI | II | 1.133 | 1.268 | 1.418 | 2.601e-04 | * |  |
| IIHHRSQKG | II | 1.091 | 1.149 | 1.210 | 1.525e-06 | * | FYPAQIKVR*, YQLELRTTL*, LGLIIHHRS*, LRTTLQRRV*, AQIKVRWFR*, VTDFYPAQI*, MLSGIGGFV*, LIIHHRSQK*, LSGIGGFVL |
| IIRQRSQKG | II | 1.033 | 1.074 | 1.117 | 0.002 | * | FRGILQRRV* |
| IIRQRSRKG | II | 1.020 | 1.066 | 1.113 | 0.022 | * | LCYFTNGTE*, LIIRQRSRK*, VYQFKGLCY*, VRGVTRHIY*, RVRGVTRHI*, YEVAYRGIL*, CRHNYEVAY |
| IIVEWRAQS | II | 1.020 | 1.059 | 1.099 | 0.015 | * |  |
| ILEQARAAV | II | 1.051 | 1.095 | 1.141 | 1.059e-04 | * |  |
| ILERKRAAV | II | 1.065 | 1.107 | 1.151 | 2.531e-06 | * | LVSRSIYNR*, RVRLVSRSI*, LLGLPAAEY*, VRLVSRSIY*, FRAVTLLGL*, EFRAVTLLG*, IVRFDSDVG*, RVCRHNYQL*, YNREEIVRF* |
| IQSMYGCDL | I | 1.414e-05 | 1.633e-05 | 1.885e-05 | 0 |  |  |
| IQVMYGCDV | I | 1.028 | 1.100 | 1.176 | 0.028 | * |  |
| ISQRKLEAA | I | 1.098 | 1.201 | 1.313 | 4.680e-04 | * |  |
| ITKRKWEAV | I | 1.054 | 1.115 | 1.180 | 0.001 | * |  |
| IVGIIAGLL | I | 1.664e-04 | 1.861e-04 | 2.081e-04 | 0 |  |  |
| IVRFDSDVG | II | 1.059 | 1.107 | 1.156 | 5.073e-05 | * | RVRLVSRSI*, LVSRSIYNR*, LLGLPAAEY*, ILERKRAAV*, EFRAVTLLG* |
| IVSGPAVLA | I | 1.063 | 1.153 | 1.251 | 0.004 | * | VVAAVIHRR*, LGIVSGPAV*, LQRAERPKT, VLGAVVAAV*, CVEWLRGYL |
| IYKAQAQTD | I | 1.047 | 1.099 | 1.154 | 0.001 | * |  |
| IYNQEENVR | II | 1.058 | 1.251 | 1.478 | 0.039 |  | LKQDKFECH |
| IYNQEEYVR | II | 1.300e-04 | 1.459e-04 | 1.637e-04 | 0 |  | NFQTLVMLE* |
| IYNREEIVR | II | 1.059 | 1.125 | 1.196 | 0.001 | * |  |
| IYNREEYAR | II | 1.067 | 1.161 | 1.263 | 0.003 | * |  |
| KAAQITQRK | I | 1.029 | 1.093 | 1.161 | 0.020 | * |  |
| KAQSQTHRV | I | 2.691e-05 | 3.081e-05 | 3.527e-05 | 0 |  |  |
| KMLSGVGGF | II | 1.890e-05 | 2.173e-05 | 2.500e-05 | 0 |  |  |
| KTGVVSTGL | II | 1.030 | 1.101 | 1.177 | 0.023 | * |  |
| KVTVYPSKS | II | 5.256e-05 | 5.967e-05 | 6.773e-05 | 0 |  |  |
| KWEAARAAE | I | 7.079e-05 | 8.005e-05 | 9.051e-05 | 0 |  |  |
| LAVLVVTVA | I | 1.142e-04 | 1.283e-04 | 1.442e-04 | 0 |  |  |
| LCYFTNGTE | II | 1.021 | 1.065 | 1.111 | 0.018 | * | VYQFKGLCY*, LIIRQRSRK*, YEVAYRGIL*, VRGVTRHIY*, IIRQRSRKG*, YRGILQRRV*, ICSVTDFYP*, CRHNYEVAY, FYPSQIKVR*, RVRGVTRHI* |
| LEAAREAEQ | I | 1.104 | 1.534 | 2.133 | 0.048 |  |  |
| LEGTCVESL | I | 1.435e-05 | 1.656e-05 | 1.912e-05 | 0 |  | TCVESLRRY*, YLEGTCVES* |
| LFYNQEEFV | II | 1.054 | 1.103 | 1.155 | 2.138e-04 | * | FYNQEEFVR*, VQFLERLFY* |
| LFYNQGEFV | II | 8.367e-05 | 9.440e-05 | 1.065e-04 | 0 |  |  |
| LGAVVAAVI | I | 1.056 | 1.194 | 1.349 | 0.023 | * |  |
| LGIVSGPAV | I | 1.065 | 1.167 | 1.279 | 0.006 | * | VVAAVIHRR*, IVSGPAVLA*, VLGAVVAAV* |
| LGLIIHHRS | II | 1.095 | 1.152 | 1.213 | 6.212e-07 | * | FYPAQIKVR*, YQLELRTTL*, LRTTLQRRV*, IIHHRSQKG*, AQIKVRWFR*, VTDFYPAQI*, MLSGIGGFV*, LIIHHRSQK*, LSGIGGFVL, GLGLIIHHR* |
| LGLIIRQRS | II | 1.065 | 1.108 | 1.152 | 3.577e-06 | * | YRAVTPQGR* |
| LGPDGRLLL | I | 7.393e-05 | 8.355e-05 | 9.442e-05 | 0 |  | LGYDQSAYD*, LLLGYDQSA* |
| LGYDQSAYD | I | 7.393e-05 | 8.355e-05 | 9.442e-05 | 0 |  | LGPDGRLLL*, LLLGYDQSA* |
| LIIHHRSQK | II | 1.061 | 1.125 | 1.192 | 6.093e-04 | * | AQIKVRWFR*, FYPAQIKVR*, YQLELRTTL*, LGLIIHHRS*, LRTTLQRRV*, LGLGLIIHH, IIHHRSQKG*, VTDFYPAQI* |
| LIIRQRSQK | II | 1.044 | 1.087 | 1.131 | 3.235e-04 | * |  |
| LIIRQRSRK | II | 1.025 | 1.065 | 1.107 | 0.008 | * | VRGVTRHIY*, VYQFKGLCY*, YEVAYRGIL*, LCYFTNGTE*, YRGILQRRV*, FYPSQIKVR*, IIRQRSRKG*, CRHNYEVAY, RVRGVTRHI*, VLEGARASV*, LICSVTDFY, VRFDSDVGV |
| LIQNGDWNF | II | 2.228e-05 | 2.558e-05 | 2.935e-05 | 0 |  |  |
| LIQNGDWTF | II | 1.039 | 1.102 | 1.168 | 0.007 | * |  |
| LKEDLRSWT | I | 1.017 | 1.067 | 1.121 | 0.039 |  |  |
| LKEDLSSWT | I | 5.572e-05 | 6.320e-05 | 7.169e-05 | 0 |  |  |
| LKGMCYFTN | II | 4.336e-05 | 4.934e-05 | 5.614e-05 | 0 |  |  |
| LLEQARAAV | II | 3.541e-06 | 4.161e-06 | 4.890e-06 | 0 |  | DLLEQARAA*, LTVEWRAWS* |
| LLERRRAAV | II | 1.085 | 1.215 | 1.360 | 0.004 | * | RVRLLERRV*, VRLLERRVH*, LVMLETVPQ*, VMLETVPQS* |
| LLFLGTGLF | II | 8.698e-06 | 1.010e-05 | 1.174e-05 | 0 |  | VQRRVQPRV, GTGLFIYFR*, TGLFIYFRN* |
| LLGLPAAEY | II | 1.065 | 1.107 | 1.150 | 2.389e-06 | * | RVRLVSRSI*, LVSRSIYNR*, EFRAVTLLG*, ILERKRAAV*, FRAVTLLGL*, VRLVSRSIY*, IVRFDSDVG* |
| LLLGYDQSA | I | 7.393e-05 | 8.355e-05 | 9.442e-05 | 0 |  | LGPDGRLLL*, LGYDQSAYD* |
| LLRGHNQNA | I | 3.280e-05 | 3.745e-05 | 4.277e-05 | 0 |  |  |
| LLRGHNQYA | I | 1.035 | 1.072 | 1.111 | 8.466e-04 | * |  |
| LLRGYNQLA | I | 1.019 | 1.080 | 1.144 | 0.040 |  |  |
| LLRGYNQYA | I | 1.505e-05 | 1.736e-05 | 2.003e-05 | 0 |  |  |
| LNEDLSSWT | I | 0.184 | 0.188 | 0.192 | 0 |  |  |
| LNHHNLLVC | II | 1.272 | 1.520 | 1.816 | 3.502e-05 | * |  |
| LQHHSLLVC | II | 1.117e-05 | 1.294e-05 | 1.498e-05 | 0 |  |  |
| LQNMYGCDL | I | 2.938e-05 | 3.360e-05 | 3.843e-05 | 0 |  |  |
| LQRMYGCDL | I | 1.020 | 1.066 | 1.114 | 0.022 | * |  |
| LQRRVEPTV | II | 1.041 | 1.098 | 1.159 | 0.004 | * |  |
| LQTRNVKAH | I | 3.385e-05 | 3.864e-05 | 4.411e-05 | 0 |  |  |
| LQWMYGCDV | I | 1.072 | 1.159 | 1.254 | 0.002 | * |  |
| LRGHDQYAY | I | 1.042 | 1.153 | 1.276 | 0.028 | * |  |
| LRGHKQYAY | I | 3.197 | 12.844 | 51.603 | 0.002 |  | LLRGHKQYA |
| LRGHNQNAY | I | 1.989e-04 | 2.220e-04 | 2.477e-04 | 0 |  |  |
| LRGYHQHAY | I | 8.660e-05 | 9.767e-05 | 1.102e-04 | 0 |  |  |
| LRGYLENGK | I | 1.067 | 1.228 | 1.414 | 0.022 | * |  |
| LRGYNQLAY | I | 1.031 | 1.128 | 1.234 | 0.040 |  |  |
| LRGYRQHAY | I | 3.707e-05 | 4.227e-05 | 4.820e-05 | 0 |  | FLRGYRQHA* |
| LRRHLENGK | I | 1.047 | 1.108 | 1.171 | 0.002 | * |  |
| LRRYLENGK | I | 1.056 | 1.140 | 1.231 | 0.005 | * |  |
| LRSWTAADK | I | 1.029 | 1.074 | 1.121 | 0.006 | * | FVQFDSDAA, WTAADKAAQ* |
| LRSWTAADV | I | 6.906e-05 | 7.812e-05 | 8.836e-05 | 0 |  | VAAQITQRK* |
| LRSWTTADK | I | 6.134e-05 | 6.949e-05 | 7.873e-05 | 0 |  | MRHFYTAVS*, WTTADKAAQ* |
| LRTTLQRRV | II | 1.106 | 1.164 | 1.225 | 6.832e-08 | * | FYPAQIKVR*, YQLELRTTL*, LGLIIHHRS*, IIHHRSQKG*, AQIKVRWFR*, VTDFYPAQI*, MLSGIGGFV*, LIIHHRSQK*, LSGIGGFVL, GLGLIIHHR* |
| LRWFRNGQE | II | 1.796e-05 | 2.067e-05 | 2.379e-05 | 0 |  | GSIELRWFR* |
| LTVEWRAWS | II | 6.694e-06 | 7.802e-06 | 9.094e-06 | 0 |  | LLEQARAAV* |
| LTVEWSARS | II | 1.033 | 1.093 | 1.157 | 0.012 | * | FLEDRRALV* |
| LVCSVTDFY | II | 1.082 | 1.159 | 1.241 | 1.841e-04 | * |  |
| LVMLETVPQ | II | 1.075 | 1.181 | 1.297 | 0.003 | * | VRLLERRVH*, RVRLLERRV*, VMLETVPQS*, LLERRRAAV* |
| LVMLETVSR | II | 8.252e-05 | 9.313e-05 | 1.051e-04 | 0 |  | NILEQARAA*, VMLETVSRS* |
| LVQNGDWTF | II | 2.746e-05 | 3.143e-05 | 3.598e-05 | 0 |  | GMVSTGLVQ*, YFYNQEENV* |
| LVRFDSDAA | I | 6.615e-05 | 7.486e-05 | 8.472e-05 | 0 |  |  |
| LVSRSIYNR | II | 1.073 | 1.115 | 1.159 | 2.533e-07 | * | RVRLVSRSI*, ILERKRAAV*, LLGLPAAEY*, FRAVTLLGL*, VRLVSRSIY*, EFRAVTLLG*, IVRFDSDVG*, RVCRHNYQL* |
| MAAEITQRK | I | 1.793e-05 | 2.064e-05 | 2.375e-05 | 0 |  |  |
| MAAQITKRK | I | 1.022 | 1.067 | 1.114 | 0.015 | * |  |
| MKAHSQTDR | I | 1.095 | 1.232 | 1.386 | 0.003 | * |  |
| MKASAQTYR | I | 1.048 | 1.106 | 1.168 | 0.002 | * |  |
| MLSGIGGFV | II | 1.053 | 1.115 | 1.181 | 0.001 | * | LSGIGGFVL, VTDFYPAQI*, LRTTLQRRV*, LGLIIHHRS*, FYPAQIKVR*, YQLELRTTL*, IIHHRSQKG* |
| MRCFYTAVS | I | 1.585e-05 | 1.827e-05 | 2.106e-05 | 0 |  |  |
| MRHFYTAVS | I | 6.134e-05 | 6.949e-05 | 7.873e-05 | 0 |  | LRSWTTADK*, WTTADKAAQ* |
| MRYFDTAVS | I | 1.011 | 1.046 | 1.083 | 0.045 |  | FDTAVSRPG |
| MRYFFTSVS | I | 1.022 | 1.063 | 1.107 | 0.013 | * | FFTSVSRPG* |
| MRYFHTAMS | I | 1.031 | 1.076 | 1.122 | 0.004 | * | YFHTAMSRP*, FHTAMSRPG* |
| MRYFYTAVS | I | 1.021 | 1.059 | 1.099 | 0.011 | * | YFYTAVSRP*, FYTAVSRPG* |
| MSPLTVEWR | II | 1.034 | 1.115 | 1.203 | 0.024 | * |  |
| MYGCDVGSD | I | 1.697e-04 | 1.897e-04 | 2.121e-04 | 0 |  |  |
| NFQTLVMLE | II | 1.300e-04 | 1.459e-04 | 1.637e-04 | 0 |  | IYNQEEYVR* |
| NILEQARAA | II | 8.252e-05 | 9.313e-05 | 1.051e-04 | 0 |  | LVMLETVSR*, VMLETVSRS* |
| NLGTLRGYY | I | 1.025 | 1.083 | 1.145 | 0.024 | * |  |
| NLRNLRGYY | I | 1.353e-05 | 1.562e-05 | 1.805e-05 | 0 |  |  |
| NMKASAQTY | I | 1.041 | 1.125 | 1.217 | 0.016 | * |  |
| NTQIYKAQA | I | 1.040 | 1.136 | 1.241 | 0.023 | * | WDRNTQIYK |
| NTQTDRESL | I | 1.561e-05 | 1.800e-05 | 2.075e-05 | 0 |  |  |
| NTRKVKAQS | I | 1.646e-05 | 1.897e-05 | 2.185e-05 | 0 |  | PTIPIVGIL*, RNTRKVKAQ* |
| NYEVAFRGI | II | 1.047 | 1.122 | 1.202 | 0.006 | * |  |
| NYQLELRTT | II | 1.032 | 1.121 | 1.218 | 0.032 | * | LIFLGLGLI, SKMLSGIGG, CSVTDFYPA |
| PAVLAVLAV | I | 1.201 | 1.525 | 1.936 | 0.003 | * | RKLEAAREA*, QRKLEAARE*, TIPNLGIVS* |
| PISDHEATL | I | 8.500e+05 | 1.018e+06 | 1.219e+06 | 0 |  | CDVGPDGRF*, HHPISDHEA*, HMTHHPISD* |
| PKMAPRAPW | I | 2.395e-05 | 2.746e-05 | 3.149e-05 | 0 |  |  |
| PLAVEWRAR | II | 1.085e-05 | 1.257e-05 | 1.456e-05 | 0 |  |  |
| PLQHHNLLV | II | 1.447e-05 | 1.670e-05 | 1.928e-05 | 0 |  |  |
| PLQHHSLLV | II | 7.415e-06 | 8.632e-06 | 1.005e-05 | 0 |  | HHSLLVCSV* |
| PRFLWQGKY | II | 9.347e-06 | 1.085e-05 | 1.259e-05 | 0 |  |  |
| PRVTVYPSK | II | 9.965e-06 | 1.156e-05 | 1.340e-05 | 0 |  | RVTVYPSKT, TGLFIYFRN* |
| PSVMSPLTV | II | 1.075 | 1.148 | 1.225 | 2.821e-04 | * |  |
| PTIPIVGIL | I | 1.646e-05 | 1.897e-05 | 2.185e-05 | 0 |  | NTRKVKAQS*, RNTRKVKAQ* |
| QETRNVKAH | I | 1.057e-05 | 1.224e-05 | 1.419e-05 | 0 |  |  |
| QIYKAQAQT | I | 1.071 | 1.142 | 1.217 | 3.696e-04 | * | YKAQAQTDR* |
| QIYKVQAQT | I | 2.113e-04 | 2.355e-04 | 2.626e-04 | 0 |  | YKVQAQTDR* |
| QLRAYLEGT | I | 1.116e-05 | 1.292e-05 | 1.496e-05 | 0 |  |  |
| QQRAYLEGR | I | 1.296e-04 | 1.454e-04 | 1.632e-04 | 0 |  |  |
| QRKLEAARE | I | 1.274 | 1.555 | 1.899 | 1.201e-04 | * | RKLEAAREA*, TIPNLGIVS*, PAVLAVLAV*, VEWLRGYLE* |
| QRKWEAARW | I | 1.176 | 1.416 | 1.705 | 0.002 | * |  |
| QRMESRAPW | I | 2.386e-06 | 2.818e-06 | 3.329e-06 | 0 |  |  |
| QRRVHPEVI | II | 8.084e-06 | 9.400e-06 | 1.093e-05 | 0 |  |  |
| QSTVPIVGI | I | 1.052 | 1.176 | 1.314 | 0.022 | * | TVPIVGIVA*, YSQAACSDS* |
| RAYLEDLCV | I | 1.977 | 3.682 | 6.858 | 3.109e-04 |  |  |
| RAYLEGGCV | I | 4.151e-05 | 4.727e-05 | 5.381e-05 | 0 |  | AYLEGGCVE* |
| RAYLEGLCV | I | 0.854 | 0.904 | 0.957 | 0.003 | * |  |
| RCFSTSVSR | I | 2.445e-04 | 2.720e-04 | 3.028e-04 | 0 |  |  |
| RFLDRHFYN | II | 1.326 | 1.685 | 2.141 | 1.596e-04 | * |  |
| RFLEYSTSE | II | 1.037 | 1.140 | 1.254 | 0.032 | * |  |
| RFLWQGKYK | II | 1.032 | 1.084 | 1.139 | 0.007 | * | FLWQGKYKC*, VQFLERLFY*, FLERLFYNQ*, YKCHFFNGT*, RVQFLERLF, FYNQEEFVR* |
| RFLWQPKRE | II | 1.026 | 1.079 | 1.135 | 0.017 | * | FLWQPKREC, PRFLWQPKR, VQRRVQPKV*, FTVQRRVQP* |
| RKLEAARAA | I | 1.029 | 1.076 | 1.126 | 0.008 | * | ARAAEQLRA |
| RKLEAAREA | I | 1.258 | 1.537 | 1.877 | 2.053e-04 | * | QRKLEAARE*, TIPNLGIVS*, PAVLAVLAV*, VEWLRGYLE* |
| RKWEAARWA | I | 1.094 | 1.235 | 1.393 | 0.004 | * | WEAARWAEQ* |
| RKWEAVHAA | I | 1.058 | 1.179 | 1.313 | 0.015 | * |  |
| RLLRGHNQN | I | 5.378e-05 | 6.102e-05 | 6.925e-05 | 0 |  |  |
| RNTQISKTN | I | 1.071e-05 | 1.241e-05 | 1.438e-05 | 0 |  |  |
| RNTRKVKAQ | I | 1.646e-05 | 1.897e-05 | 2.185e-05 | 0 |  | NTRKVKAQS*, PTIPIVGIL* |
| RRVEPTVTI | II | 8.068e-06 | 9.381e-06 | 1.091e-05 | 0 |  | LSGVGGFVL |
| RSHTLQRMF | I | 1.931e-05 | 2.220e-05 | 2.552e-05 | 0 |  |  |
| RVCRHNYQL | II | 1.038 | 1.089 | 1.143 | 0.003 | * | ILERKRAAV*, LVSRSIYNR* |
| RVRGVTRHI | II | 1.038 | 1.087 | 1.138 | 0.003 | * | VLEGARASV*, YEVAYRGIL*, VYQFKGLCY*, IIRQRSRKG*, VRGVTRHIY*, LIIRQRSRK*, ALNHHNLLI, LCYFTNGTE* |
| RVRLLERRV | II | 1.091 | 1.211 | 1.346 | 0.002 | * | LVMLETVPQ*, VRLLERRVH*, LLERRRAAV*, VMLETVPQS* |
| RVRLVSRSI | II | 1.072 | 1.113 | 1.156 | 2.831e-07 | * | LVSRSIYNR*, LLGLPAAEY*, ILERKRAAV*, EFRAVTLLG*, VRLVSRSIY*, FRAVTLLGL*, IVRFDSDVG* |
| RVRLVTRYI | II | 1.014 | 1.051 | 1.090 | 0.031 | * | VRLVTRYIY*, LVTRYIYNR |
| RVSLRNLRG | I | 1.017 | 1.063 | 1.111 | 0.031 | * |  |
| RVYLEGRCV | I | 1.068 | 1.120 | 1.175 | 2.816e-05 | * | VHAAEQRRV* |
| RYFYTSMSR | I | 1.045 | 1.148 | 1.262 | 0.021 | * | YTSMSRPGR*, MRYFYTSMS, FYTSMSRPG |
| SESAQSKML | II | 1.287 | 1.291 | 1.296 | 0 |  |  |
| SFTVQRRVH | II | 1.049 | 1.100 | 1.153 | 5.957e-04 | * |  |
| SFTVQRRVQ | II | 1.057 | 1.104 | 1.154 | 8.650e-05 | * | FTVQRRVQP*, VQRRVQPKV* |
| SGLQPTGFL | II | 8.204e-07 | 9.825e-07 | 1.176e-06 | 0 |  |  |
| SHTIQIMHG | I | 1.646 | 4.394 | 11.731 | 0.017 |  |  |
| SHTLQMMYG | I | 1.726e-05 | 1.988e-05 | 2.289e-05 | 0 |  |  |
| SHTVQRMFG | I | 3.079e-05 | 3.519e-05 | 4.022e-05 | 0 |  | VQRMFGCDV* |
| SIEVRWFWN | II | 1.111e-04 | 1.249e-04 | 1.404e-04 | 0 |  | EVRWFWNGQ*, WFWNGQEEK* |
| SIYNREEIV | II | 1.107 | 1.202 | 1.306 | 1.086e-04 | * |  |
| SLLVCSVSG | II | 5.138e-05 | 5.834e-05 | 6.624e-05 | 0 |  |  |
| SMRCFSTSV | I | 1.017e-05 | 1.179e-05 | 1.367e-05 | 0 |  |  |
| SQIDRVDLG | I | 0.632 | 0.635 | 0.639 | 0 |  |  |
| SQIKVRWFR | II | 1.016 | 1.067 | 1.121 | 0.042 |  | FYPSQIKVR* |
| SQKDLLERR | II | 6.705e-06 | 7.815e-06 | 9.109e-06 | 0 |  |  |
| SQPLQHHNL | II | 3.537e-05 | 4.035e-05 | 4.604e-05 | 0 |  |  |
| SSQSTVPIV | I | 1.082 | 1.275 | 1.502 | 0.019 | * |  |
| SVTDFYPSH | II | 0.626 | 0.629 | 0.633 | 0 |  |  |
| SVTDFYPSQ | II | 9.611e-05 | 1.082e-04 | 1.219e-04 | 0 |  |  |
| TCVESLRRY | I | 2.156e-05 | 2.476e-05 | 2.842e-05 | 0 |  | YLEGTCVES*, LEGTCVESL* |
| TCVEWLRRH | I | 1.061 | 1.140 | 1.226 | 0.002 | * |  |
| TDRESLRTL | I | 3.016e-06 | 3.551e-06 | 4.182e-06 | 0 |  |  |
| TDRVDLATL | I | 2.617e-05 | 2.997e-05 | 3.433e-05 | 0 |  |  |
| TDRVGLRNL | I | 2.523e-06 | 2.978e-06 | 3.515e-06 | 0 |  | ECVEWLRRH |
| TGLFIYFRN | II | 9.440e-06 | 1.095e-05 | 1.271e-05 | 0 |  | LLFLGTGLF*, PRVTVYPSK*, VQRRVQPRV |
| TGVVSTPLI | II | 1.025 | 1.097 | 1.173 | 0.033 | * |  |
| TIPNLGIVS | I | 1.213 | 1.441 | 1.714 | 2.681e-04 | * | QRKLEAARE*, RKLEAAREA*, PAVLAVLAV* |
| TLLGLPAAE | II | 1.042 | 1.108 | 1.178 | 0.006 | * |  |
| TLLGLPDAE | II | 5.249e-05 | 5.958e-05 | 6.763e-05 | 0 |  |  |
| TQISKTNTQ | I | 4.885e-06 | 5.717e-06 | 6.690e-06 | 0 |  |  |
| TQPLQHHSL | II | 1.516e-05 | 1.748e-05 | 2.016e-05 | 0 |  |  |
| TQTYRENLR | I | 1.028 | 1.125 | 1.231 | 0.045 |  |  |
| TRNMKAHSQ | I | 1.035 | 1.120 | 1.212 | 0.025 | * | KRKWEAVHA, FDSDAASQK |
| TRNMKASAQ | I | 1.053 | 1.189 | 1.343 | 0.026 | * |  |
| TTGVVSTPL | II | 1.030 | 1.087 | 1.147 | 0.012 | * | TVCRHNYQL |
| TVHIVGIIA | I | 1.053 | 1.175 | 1.311 | 0.020 | * |  |
| TVPIVGIVA | I | 1.084 | 1.190 | 1.306 | 0.002 | * | QSTVPIVGI* |
| TYRESLRIA | I | 3.139e-05 | 3.586e-05 | 4.098e-05 | 0 |  |  |
| TYRESLRTA | I | 8.554e-06 | 9.939e-06 | 1.155e-05 | 0 |  |  |
| VAAQITQRK | I | 6.906e-05 | 7.812e-05 | 8.836e-05 | 0 |  | LRSWTAADV* |
| VAAVMWRRK | I | 1.037 | 1.104 | 1.175 | 0.011 | * |  |
| VAHVAEQLR | I | 5.668e-05 | 6.428e-05 | 7.289e-05 | 0 |  |  |
| VCSVSGFYP | II | 1.108 | 1.304 | 1.536 | 0.008 | * |  |
| VCSVTDFYP | II | 4.517e-06 | 5.292e-06 | 6.199e-06 | 0 |  |  |
| VDTVCRHNY | II | 1.024 | 1.107 | 1.197 | 0.048 |  |  |
| VESLRRYLE | I | 2.136 | 2.157 | 2.179 | 0 |  |  |
| VEWLRGYLE | I | 1.097 | 1.303 | 1.548 | 0.014 | * | QRKLEAARE*, RKLEAAREA* |
| VFQFKGMCY | II | 1.056 | 1.100 | 1.147 | 5.158e-05 | * |  |
| VGGFMLGLL | II | 1.906e-05 | 2.192e-05 | 2.520e-05 | 0 |  | FMLGLLFLG*, GFMLGLLFL* |
| VGYVDDSQF | I | 1.931e-05 | 2.220e-05 | 2.553e-05 | 0 |  |  |
| VHAAEQRRV | I | 1.044 | 1.087 | 1.132 | 4.088e-04 | * | RVYLEGRCV* |
| VHPEVTVYP | II | 1.109 | 1.248 | 1.406 | 0.002 | * | CQVEHPSVM* |
| VIGAVVAAV | I | 1.035 | 1.083 | 1.133 | 0.004 | * | VVAAVMCRR* |
| VIVYPAKTQ | II | 6.402e-06 | 7.466e-06 | 8.708e-06 | 0 |  |  |
| VLAVLAVLA | I | 1.069 | 1.223 | 1.400 | 0.018 | * |  |
| VLEGARASV | II | 1.028 | 1.076 | 1.127 | 0.010 | * | ALNHHNLLI, RVRGVTRHI*, CRHNYEVAY, VRGVTRHIY*, LIIRQRSRK* |
| VLERTRAAV | II | 1.440e-05 | 1.662e-05 | 1.919e-05 | 0 |  |  |
| VLGAVVAAV | I | 1.051 | 1.168 | 1.298 | 0.020 | * | LGIVSGPAV*, VVAAVIHRR*, IVSGPAVLA* |
| VLVVTVAVV | I | 4.570e-06 | 5.353e-06 | 6.271e-06 | 0 |  |  |
| VMAVVMCRR | I | 1.034 | 1.068 | 1.104 | 6.337e-04 | * |  |
| VMCRRKSSG | I | 1.189 | 1.191 | 1.194 | 0 |  |  |
| VMLETVPQS | II | 1.087 | 1.206 | 1.337 | 0.003 | * | LVMLETVPQ*, VRLLERRVH*, LLERRRAAV*, RVRLLERRV* |
| VMLETVPRS | II | 0.890 | 0.892 | 0.893 | 0 |  |  |
| VMLETVSRS | II | 8.252e-05 | 9.313e-05 | 1.051e-04 | 0 |  | LVMLETVSR*, NILEQARAA* |
| VQFLERLFY | II | 1.025 | 1.072 | 1.122 | 0.014 | * | RFLWQGKYK*, FYNQEEFVR*, FLWQGKYKC*, FLERLFYNQ*, LFYNQEEFV*, YKCHFFNGT* |
| VQFLESLFY | II | 8.820e-05 | 9.945e-05 | 1.121e-04 | 0 |  | FLESLFYNQ* |
| VQRMFGCDV | I | 4.652e-05 | 5.289e-05 | 6.013e-05 | 0 |  | SHTVQRMFG* |
| VQRRVHPEV | II | 1.079 | 1.144 | 1.213 | 5.525e-05 | * |  |
| VQRRVHPKV | II | 1.096 | 1.153 | 1.212 | 3.024e-07 | * |  |
| VQRRVQPKV | II | 1.064 | 1.110 | 1.158 | 1.036e-05 | * | FTVQRRVQP*, RFLWQPKRE*, SFTVQRRVQ* |
| VQWFRNDQE | II | 2.744e-05 | 3.141e-05 | 3.595e-05 | 0 |  |  |
| VRFDSDATS | I | 1.016 | 1.068 | 1.123 | 0.045 |  |  |
| VRFLDRHFY | II | 1.164 | 1.279 | 1.404 | 2.510e-06 | * |  |
| VRFLDRYFY | II | 1.019 | 1.065 | 1.113 | 0.025 | * |  |
| VRGVTRHIY | II | 1.022 | 1.062 | 1.104 | 0.013 | * | LIIRQRSRK*, YEVAYRGIL*, VYQFKGLCY*, LCYFTNGTE*, FYPSQIKVR*, YRGILQRRV*, CRHNYEVAY, IIRQRSRKG*, RVRGVTRHI*, VLEGARASV*, VRFDSDVGV |
| VRLLERRVH | II | 1.068 | 1.182 | 1.310 | 0.008 | * | LVMLETVPQ*, RVRLLERRV*, LLERRRAAV*, VMLETVPQS* |
| VRLVSRSIY | II | 1.060 | 1.105 | 1.151 | 1.924e-05 | * | ILERKRAAV*, LVSRSIYNR*, RVRLVSRSI*, LLGLPAAEY* |
| VRLVTRYIY | II | 1.016 | 1.054 | 1.093 | 0.023 | * | RVRLVTRYI*, LVTRYIYNR |
| VRWFWNGQE | II | 3.331e-05 | 3.804e-05 | 4.343e-05 | 0 |  |  |
| VRYLDRYFH | II | 1.027 | 1.077 | 1.128 | 0.012 | * |  |
| VRYLHRGIY | II | 1.031 | 1.130 | 1.239 | 0.041 |  | RVRYLHRGI, YLHRGIYNQ |
| VTDFYPAQI | II | 1.059 | 1.122 | 1.189 | 6.492e-04 | * | MLSGIGGFV*, LSGIGGFVL, LRTTLQRRV*, LGLIIHHRS*, FYPAQIKVR*, YQLELRTTL*, IIHHRSQKG*, LGLGLIIHH, AQIKVRWFR*, LIIHHRSQK* |
| VTELGRPVA | II | 1.081 | 1.172 | 1.271 | 9.032e-04 | * |  |
| VTLLGLPAA | II | 1.028 | 1.082 | 1.138 | 0.013 | * |  |
| VTRYIYNRE | II | 1.043 | 1.103 | 1.166 | 0.004 | * |  |
| VTVYPAKTQ | II | 1.022 | 1.077 | 1.134 | 0.025 | * |  |
| VVAAVIHRR | I | 1.063 | 1.154 | 1.252 | 0.004 | * | IVSGPAVLA*, LGIVSGPAV*, LQRAERPKT, VLGAVVAAV*, CVEWLRGYL |
| VVAAVMCRR | I | 1.051 | 1.093 | 1.137 | 6.890e-05 | * | VIGAVVAAV* |
| VVCRRKSSG | I | 9.847e-06 | 1.142e-05 | 1.325e-05 | 0 |  |  |
| VVMCRRKSS | I | 1.043 | 1.106 | 1.173 | 0.004 | * |  |
| VVSTGLIRN | II | 4.215e-05 | 4.798e-05 | 5.462e-05 | 0 |  |  |
| VVVPSGQEQ | I | 4.524 | 4.615 | 4.708 | 0 |  |  |
| VYLEGRCVD | I | 1.041 | 1.142 | 1.252 | 0.025 | * |  |
| VYPAKTQPL | II | 1.044 | 1.086 | 1.130 | 3.695e-04 | * |  |
| VYPSKSQPL | II | 1.612e-05 | 1.858e-05 | 2.141e-05 | 0 |  | HAGLQPTGF* |
| VYPSKTQPL | II | 1.131 | 1.216 | 1.308 | 1.290e-06 | * |  |
| VYQFKGLCY | II | 1.027 | 1.070 | 1.116 | 0.008 | * | YEVAYRGIL*, LCYFTNGTE*, LIIRQRSRK*, VRGVTRHIY*, RVRGVTRHI*, IIRQRSRKG*, YRGILQRRV*, ICSVTDFYP*, YVRFDSDVG*, LICSVTDFY, FYPSQIKVR* |
| VYQFKGMCY | II | 1.033 | 1.078 | 1.124 | 0.003 | * | FVYQFKGMC* |
| VYRAVTPLG | II | 1.031 | 1.073 | 1.117 | 0.003 | * | TVCRHNYQL |
| VYRAVTPQG | II | 1.039 | 1.084 | 1.131 | 0.001 | * |  |
| WAEQLRAYL | I | 1.068 | 1.156 | 1.251 | 0.002 | * |  |
| WDGETRNMK | I | 7.856e-05 | 8.872e-05 | 1.002e-04 | 0 |  |  |
| WDQETRKVK | I | 8.313e-05 | 9.380e-05 | 1.058e-04 | 0 |  |  |
| WDQETRNMK | I | 1.145 | 1.352 | 1.596 | 0.002 | * |  |
| WEAAHVAEQ | I | 1.038 | 1.087 | 1.139 | 0.003 | * |  |
| WEAARRAEQ | I | 1.036 | 1.149 | 1.274 | 0.039 |  | RAEQLRAYL, QRKWEAARR, ARRAEQLRA* |
| WEAARWAEQ | I | 1.092 | 1.245 | 1.420 | 0.006 | * | RKWEAARWA* |
| WEAVHAAEQ | I | 1.036 | 1.090 | 1.146 | 0.005 | * |  |
| WEPSSQSTV | I | 1.060 | 1.150 | 1.248 | 0.005 | * |  |
| WFRKGQEEK | II | 8.875e-05 | 1.001e-04 | 1.128e-04 | 0 |  | FRKGQEEKT* |
| WFWNGQEEK | II | 1.111e-04 | 1.249e-04 | 1.404e-04 | 0 |  | EVRWFWNGQ*, SIEVRWFWN* |
| WNFQTLVML | II | 3.346e-05 | 3.820e-05 | 4.361e-05 | 0 |  |  |
| WNSQKDFLE | II | 5.840e-05 | 6.620e-05 | 7.504e-05 | 0 |  |  |
| WQRMYGCDL | I | 1.037 | 1.120 | 1.210 | 0.020 | * |  |
| WRAWSESAQ | II | 9.601e-06 | 1.114e-05 | 1.292e-05 | 0 |  | WSESAQSKM* |
| WRFLRGYHQ | I | 1.014 | 1.050 | 1.086 | 0.027 | * | DWRFLRGYH, FGAVITGAV*, VKAHSQTHR |
| WSESAQSKM | II | 9.601e-06 | 1.114e-05 | 1.292e-05 | 0 |  | WRAWSESAQ* |
| WTAADKAAQ | I | 1.021 | 1.078 | 1.137 | 0.031 | * | LRSWTAADK* |
| WTTADKAAQ | I | 6.134e-05 | 6.949e-05 | 7.873e-05 | 0 |  | LRSWTTADK*, MRHFYTAVS* |
| YCRHNYGVV | II | 1.056 | 1.105 | 1.157 | 1.497e-04 | * | YGVVESFTV* |
| YEVAFRGIL | II | 1.022 | 1.064 | 1.108 | 0.014 | * | FYPGQIKVR*, FRGILQRRV*, CRHNYEVAF |
| YEVAYRGIL | II | 1.029 | 1.073 | 1.118 | 0.006 | * | VYQFKGLCY*, LIIRQRSRK*, VRGVTRHIY*, LCYFTNGTE*, RVRGVTRHI*, IIRQRSRKG*, YRGILQRRV*, FYPSQIKVR*, YVRFDSDVG* |
| YFFTSVSRP | I | 1.034 | 1.078 | 1.124 | 0.003 | * | FFTSVSRPG* |
| YFHNQEESV | II | 3.839e-05 | 4.376e-05 | 4.987e-05 | 0 |  |  |
| YFHTAMSRP | I | 1.035 | 1.080 | 1.126 | 0.003 | * | MRYFHTAMS*, FHTAMSRPG* |
| YFRNQKGHA | II | 4.516e-06 | 5.291e-06 | 6.198e-06 | 0 |  |  |
| YFYNQEEFV | II | 1.014e-05 | 1.176e-05 | 1.364e-05 | 0 |  |  |
| YFYNQEENV | II | 2.746e-05 | 3.143e-05 | 3.598e-05 | 0 |  | GMVSTGLVQ*, LVQNGDWTF* |
| YFYNQEEYV | II | 1.018 | 1.064 | 1.111 | 0.027 | * | FYNQEEYVR |
| YFYTAVSRP | I | 1.031 | 1.070 | 1.110 | 0.002 | * | MRYFYTAVS*, FYTAVSRPG* |
| YGVVESFTV | II | 1.051 | 1.098 | 1.146 | 1.793e-04 | * | YCRHNYGVV* |
| YIALKEDLR | I | 1.052 | 1.092 | 1.133 | 3.081e-05 | * |  |
| YIALKEDLS | I | 1.929e-05 | 2.218e-05 | 2.550e-05 | 0 |  |  |
| YIYNREEYA | II | 1.060 | 1.106 | 1.153 | 2.459e-05 | * |  |
| YIYNREEYV | II | 2.173e-06 | 2.570e-06 | 3.039e-06 | 0 |  |  |
| YKAQAQTDR | I | 1.054 | 1.119 | 1.187 | 0.001 | * | QIYKAQAQT* |
| YKCHFFNGT | II | 1.017 | 1.069 | 1.124 | 0.037 |  | FLWQGKYKC*, FLERLFYNQ*, RFLWQGKYK*, FYNQEEFVR*, VQFLERLFY*, FFNGTERVQ* |
| YKRQAQTDR | I | 1.044 | 1.095 | 1.148 | 0.001 | * |  |
| YKVQAQTDR | I | 2.113e-04 | 2.355e-04 | 2.626e-04 | 0 |  | QIYKVQAQT* |
| YLEGTCVDW | I | 4.227e-05 | 4.811e-05 | 5.476e-05 | 0 |  |  |
| YLEGTCVES | I | 2.156e-05 | 2.476e-05 | 2.842e-05 | 0 |  | TCVESLRRY*, LEGTCVESL* |
| YLENGKKTL | I | 1.017 | 1.060 | 1.104 | 0.027 | * |  |
| YNQEESVRF | II | 1.042 | 1.086 | 1.131 | 6.116e-04 | * |  |
| YNQEETVRF | II | 9.780e-05 | 1.101e-04 | 1.240e-04 | 0 |  |  |
| YNREEIVRF | II | 1.053 | 1.103 | 1.155 | 2.639e-04 | * | ILERKRAAV* |
| YQFKAMCYF | II | 1.187 | 1.421 | 1.700 | 9.082e-04 | * |  |
| YQLELRTTL | II | 1.095 | 1.153 | 1.213 | 5.700e-07 | * | FYPAQIKVR*, LGLIIHHRS*, LRTTLQRRV*, IIHHRSQKG*, AQIKVRWFR*, VTDFYPAQI*, MLSGIGGFV*, LIIHHRSQK*, LSGIGGFVL, GLGLIIHHR* |
| YRAVTELGR | II | 1.015 | 1.066 | 1.120 | 0.045 |  |  |
| YRAVTPLGQ | II | 8.406e-06 | 9.769e-06 | 1.135e-05 | 0 |  |  |
| YRAVTPQGR | II | 1.066 | 1.108 | 1.152 | 1.946e-06 | * | LGLIIRQRS* |
| YRGILQRRV | II | 1.023 | 1.062 | 1.103 | 0.008 | * | LIIRQRSRK*, VRGVTRHIY*, VYQFKGLCY*, LCYFTNGTE*, YEVAYRGIL* |
| YSQAACSDS | I | 1.043 | 1.160 | 1.291 | 0.029 | * | QSTVPIVGI* |
| YSQAAYSDS | I | 1.478e-05 | 1.705e-05 | 1.967e-05 | 0 |  |  |
| YSQAVSSDS | I | 8.675e-05 | 9.784e-05 | 1.103e-04 | 0 |  |  |
| YSTSECHFF | II | 1.093 | 1.175 | 1.264 | 1.118e-04 | * |  |
| YTAVSRPGR | I | 1.055 | 1.102 | 1.150 | 8.581e-05 | * |  |
| YTCHVQHEG | I | 2.344e-05 | 2.689e-05 | 3.084e-05 | 0 |  |  |
| YTSMSRPGR | I | 1.045 | 1.148 | 1.260 | 0.020 | * | RYFYTSMSR*, MRYFYTSMS, FYTSMSRPG |
| YTSVSRPGS | I | 3.214e-05 | 3.671e-05 | 4.194e-05 | 0 |  |  |
| YVRFDSDVG | II | 1.014 | 1.055 | 1.097 | 0.037 |  | YEVAYRGIL*, VYQFKGLCY* |
| YWDQETRKV | I | 5.270e-06 | 6.162e-06 | 7.204e-06 | 0 |  |  |
| YYNQSEDRS | I | 9.828e-06 | 1.140e-05 | 1.322e-05 | 0 |  |  |

HR: hazard ratio; LB: Lower bound of the 95% confidence interval (95% CI); UB: Upper bound of the 95% CI

^a^ Subset of TcEMM represented in Figure 2 (present in ≥50 donor:recipient pairs and statistically significantly associated with DCGF in AFT models accounting for multiple testing)

^b^Highly correlated TcEMMs (correlation > 0.74) are listed sequentially by descending degree of correlation with the TcEMM of interest.

*Highly correlated TcEMMs also included among the TcEMM individually associated with DCGF in AFT models accounting for multiple testing.
