# Supplementary material for: Dissecting the impact of molecular T-cell HLA mismatches in kidney transplant failure: A retrospective cohort study
Source: Front Immunol. 2022 Nov 24;13:1067075. doi: 10.3389/fimmu.2022.1067075 (PMC9730505; doi:10.3389/fimmu.2022.1067075)
Supplement: Supplementary file 5 [file Table_4.docx]

**Supplementary Table 4. TcEMMs predictive of death-censored graft failure in Lasso penalized regression models**

| TcEMM | HLA Class | Single Variable Regression | | | HR by Penalized Regression | Post-Selection Inference  (p-value) | Previously Identified^a^ | Highly-correlated coexpressed TcEMMs^b^ |
| --- | --- | --- | --- | --- | --- | --- | --- | --- |
|  |  | **LB** | **HR** | **UB** |  |  |  |  |
| AAEQQRAYL | I | 0.787 | 0.895 | 1.019 | 0.995 | 0.984 |  |  |
| AAQITKRKW | I | 1.040 | 1.129 | 1.226 | 1.032 | 0.993 |  |  |
| AARAAEQLR | I | 0.739 | 0.872 | 1.030 | 0.978 | 0.038 |  |  |
| AASQKMEPR | I | 1.103 | 1.639 | 2.434 | 1.119 | 0.052 |  | TKRKWEAVH |
| AELDTVCRH | II | 1.019 | 1.090 | 1.165 | 1.004 | 0.969 |  |  |
| AGSHIIQRM | I | 0.950 | 1.128 | 1.340 | 1.045 | 0.027 |  |  |
| AGSHILQRM | I | 0.898 | 1.927 | 4.133 | 1.071 | 0.015 |  |  |
| AGSHTLQWM | I | 3.392e-06 | 3.989e-06 | 4.690e-06 | 0.394 | 0.983 |  |  |
| AGSHTVQRM | I | 1.111 | 5.231 | 24.638 | 1.268 | 0.995 | ◊ |  |
| ALGFYPTEI | I | 0.983 | 1.089 | 1.206 | 1.016 | 0.027 |  |  |
| ALSFYPAEI | I | 0.778 | 0.913 | 1.071 | 0.956 | 0.063 |  |  |
| AQADRVNLR | I | 0.987 | 1.044 | 1.104 | 1.011 | 0.017 |  |  |
| AQIKVRWFR | II | 1.087 | 1.147 | 1.212 | 1.033 | 0.997 |  | LRTTLQRRV*, FYPAQIKVR, YQLELRTTL, LGLIIHHRS, LIIHHRSQK, IIHHRSQKG, VTDFYPAQI |
| ARRAEQLRA | I | 1.044 | 1.162 | 1.293 | 1.025 | 0.996 |  | RAEQLRAYL, QRKWEAARR, WEAARRAEQ* |
| ARWAEQLRA | I | 1.210 | 1.619 | 2.165 | 1.193 | 0.953 |  |  |
| ATLRCWALS | I | 1.062 | 1.235 | 1.435 | 1.014 | 0.081 |  |  |
| AVVAAVMWR | I | 0.527 | 0.688 | 0.899 | 0.808 | 0.724 |  |  |
| AVVMCRRKS | I | 1.047 | 1.126 | 1.212 | 1.030 | 0.057 |  |  |
| CDVGPDGRF | I | 8.500e+05 | 1.018e+06 | 1.219e+06 | 2.757 | 0.015 |  | HHPISDHEA*, HMTHHPISD, PISDHEATL |
| CFYTAVSRP | I | 1.160 | 8.259 | 58.800 | 3.204 | 0.018 |  |  |
| CHFFNGTEG | II | 9.089 | 64.770 | 461.566 | 15.768 | 0.318 |  | FFNGTEGVQ*, FNGTEGVQF*, GVQFLERLF* |
| CHMQHEGLQ | I | 0.466 | 0.734 | 1.157 | 0.920 | 0.958 |  |  |
| CHVEHPSLQ | II | 0.622 | 0.859 | 1.185 | 0.995 | 0.925 |  |  |
| DILEDKRAA | II | 1.000 | 2.048 | 4.194 | 1.297 | 0.997 |  |  |
| DLATLRGYY | I | 1.346 | 3.244 | 7.814 | 1.943 | 0.121 |  |  |
| DLLEDRRAA | II | 0.793 | 1.617 | 3.297 | 1.065 | 0.038 |  |  |
| DRESLRTLL | I | 1.443e-06 | 1.715e-06 | 2.039e-06 | 0.843 | 0.008 |  |  |
| DRVSLRNLR | I | 1.009 | 1.061 | 1.115 | 1.027 | 0.020 |  |  |
| DVGPDGRLL | I | 1.053 | 1.356 | 1.746 | 1.124 | 0.032 |  |  |
| EAEQLRAYL | I | 1.039 | 1.122 | 1.212 | 1.011 | 0.034 |  |  |
| ERVRLVTRH | II | 1.030 | 1.141 | 1.263 | 1.021 | 0.995 |  |  |
| ERVRLVTRY | II | 0.900 | 0.968 | 1.041 | 0.997 | 0.914 |  |  |
| EVAYRGILQ | II | 0.090 | 0.412 | 1.882 | 0.850 | 0.019 |  |  |
| EWRAQSEPA | II | 1.025 | 4.108 | 16.459 | 1.759 | 0.009 |  |  |
| FAGAVVAAV | I | 0.983 | 1.025 | 1.069 | 1.002 | 0.003 |  | VVAAVRWRR, FGAVFAGAV, YFTTSVSRP |
| FDSDVGEFR | II | 1.030 | 1.076 | 1.125 | 1.001 | 0.012 |  |  |
| FDSDVGVYR | II | 1.057 | 1.104 | 1.154 | 1.021 | 0.035 |  |  |
| FDTAVSRPS | I | 5.552e-06 | 6.487e-06 | 7.579e-06 | 0.874 | 0.980 |  |  |
| FFNGTEGVQ | II | 9.089 | 64.770 | 461.566 | 1.003 | 0.475 |  | CHFFNGTEG*, FNGTEGVQF*, GVQFLERLF* |
| FFTSVSRPG | I | 1.037 | 1.076 | 1.117 | 1.016 | 0.042 |  | MRYFFTSVS, YFFTSVSRP* |
| FLEYSTSEC | II | 1.089 | 1.140 | 1.193 | 1.020 | 0.964 |  |  |
| FLGLGLIIR | II | 0.863 | 0.965 | 1.079 | 0.998 | 0.031 | ◊ |  |
| FLRGYHQYA | I | 1.037 | 1.073 | 1.110 | 1.027 | 0.974 | ◊ |  |
| FNGTEGVQF | II | 9.089 | 64.770 | 461.566 | 1.001 | 0.906 |  | CHFFNGTEG*, FFNGTEGVQ*, GVQFLERLF* |
| FNGTERVRL | II | 1.026 | 1.068 | 1.112 | 1.032 | 0.995 |  | VRLLERCIY |
| FQNGQEEKA | II | 0.533 | 3.795 | 27.012 | 1.071 | 0.056 |  | VCRHNYGVV, VRWFQNGQE |
| FTSVSRPGR | I | 1.029 | 1.080 | 1.133 | 1.012 | 0.926 |  |  |
| FTVQRRVQP | II | 1.068 | 1.113 | 1.159 | 1.029 | 0.017 |  | SFTVQRRVQ, VQRRVQPKV*, RFLWQPKRE |
| FVLGLIFLG | II | 0.105 | 1.688 | 27.075 | 1.163 | 0.991 |  |  |
| FYTAVSRPG | I | 1.044 | 1.081 | 1.119 | 1.007 | 0.993 |  | YFYTAVSRP*, MRYFYTAVS |
| FYTSVSRPG | I | 1.000 | 1.037 | 1.074 | 1.010 | 0.022 |  | YFYTSVSRP, MRYFYTSVS |
| GFYPTEITL | I | 1.244e-05 | 1.438e-05 | 1.663e-05 | 0.996 | 0.991 |  |  |
| GLAVLAVLA | I | 1.082 | 1.221 | 1.379 | 1.040 | 0.919 |  |  |
| GLFIYFRNQ | II | 1.738 | 12.363 | 87.972 | 3.894 | 0.123 |  |  |
| GMCYFTNGT | II | 0.637 | 0.829 | 1.079 | 0.981 | 0.132 |  |  |
| GQIKVQWFR | II | 1.026 | 1.203 | 1.411 | 1.019 | 0.981 |  | VGYRGILQR |
| GRLLLGYDQ | I | 26.764 | 190.796 | 1.360e+03 | 43.095 | 0.064 |  |  |
| GRLLRGYNR | I | 0.837 | 5.969 | 42.560 | 1.720 | 3.036e-04 |  | LLRGYNRLA*, LRGYNRLAY*, RLLRGYNRL*, YNRLAYDGK*, YTCYVQHEG*, YVQHEGLPK* |
| GTQFVRFDS | I | 0.593 | 0.792 | 1.059 | 0.995 | 0.009 |  |  |
| GVQFLERLF | II | 9.089 | 64.770 | 461.566 | 1.005 | 0.344 |  | CHFFNGTEG*, FFNGTEGVQ*, FNGTEGVQF* |
| HHPISDHEA | I | 8.500e+05 | 1.018e+06 | 1.219e+06 | 1.002 | 0.984 |  | CDVGPDGRF*, HMTHHPISD, PISDHEATL |
| HILQRMYGC | I | 1.218 | 1.727 | 2.449 | 1.061 | 0.948 |  |  |
| HIYNREEYA | II | 0.992 | 1.225 | 1.513 | 1.019 | 0.982 |  |  |
| HLENGKETL | I | 1.051 | 1.313 | 1.641 | 1.008 | 0.936 |  |  |
| HMTHHAVSD | I | 1.408 | 2.390 | 4.054 | 1.197 | 0.006 |  |  |
| HVAEQRRAY | I | 1.019 | 7.247 | 51.566 | 2.170 | 0.015 |  |  |
| IALKEDLRS | I | 1.037 | 1.120 | 1.210 | 1.053 | 0.985 |  |  |
| IGAVVAAVM | I | 1.086 | 1.152 | 1.223 | 1.015 | 0.977 |  |  |
| IGGFVLGLI | II | 1.133 | 1.268 | 1.418 | 1.025 | 0.025 |  |  |
| IIVEWRAQS | II | 1.020 | 1.059 | 1.099 | 1.025 | 0.015 |  |  |
| ILEDKRAAV | II | 1.004 | 1.115 | 1.239 | 1.032 | 0.976 |  |  |
| ILEEDRASV | II | 0.984 | 1.058 | 1.138 | 1.000 | 0.994 |  |  |
| ISQRKLEAA | I | 1.098 | 1.201 | 1.313 | 1.030 | 0.001 |  |  |
| ITLTWQWDG | I | 1.033 | 1.571 | 2.391 | 1.222 | 0.968 |  |  |
| IVSGPAVLA | I | 1.063 | 1.153 | 1.251 | 1.000 | 0.014 |  | VVAAVIHRR, LGIVSGPAV, LQRAERPKT, VLGAVVAAV, CVEWLRGYL |
| IYKAQAQTD | I | 1.047 | 1.099 | 1.154 | 1.027 | 0.117 |  |  |
| IYNQEENVR | II | 1.058 | 1.251 | 1.478 | 1.071 | 0.971 |  | LKQDKFECH |
| IYNREEYAR | II | 1.067 | 1.161 | 1.263 | 1.027 | 0.035 |  |  |
| KAGVVSTGL | II | 1.312 | 7.888 | 47.431 | 4.788 | 0.150 |  |  |
| LAVLAVLAV | I | 0.560 | 0.752 | 1.010 | 0.905 | 0.008 |  |  |
| LAYDGKDYI | I | 1.009 | 1.079 | 1.154 | 1.001 | 0.025 |  |  |
| LEGECVEWL | I | 1.001 | 1.118 | 1.249 | 1.010 | 0.011 |  |  |
| LERTRAELD | II | 0.844 | 0.925 | 1.013 | 0.936 | 0.018 |  |  |
| LFGAVITGA | I | 0.346 | 2.074 | 12.430 | 1.266 | 0.987 |  |  |
| LFYNQEEFV | II | 1.054 | 1.103 | 1.155 | 1.022 | 0.984 |  | FYNQEEFVR, VQFLERLFY |
| LIIRQRSQK | II | 1.044 | 1.087 | 1.131 | 1.038 | 0.987 |  |  |
| LIQNGDWTF | II | 1.039 | 1.102 | 1.168 | 1.066 | 0.011 |  |  |
| LLERRRAAV | II | 1.085 | 1.215 | 1.360 | 1.000 | 0.979 |  | RVRLLERRV*, VRLLERRVH, LVMLETVPQ, VMLETVPQS* |
| LLRGHNQYA | I | 1.035 | 1.072 | 1.111 | 1.024 | 0.025 |  |  |
| LLRGYDQSA | I | 0.872 | 0.935 | 1.002 | 0.965 | 0.046 |  |  |
| LLRGYNRLA | I | 0.837 | 5.969 | 42.560 | 1.016 | 0.006 |  | GRLLRGYNR*, LRGYNRLAY*, RLLRGYNRL*, YNRLAYDGK*, YTCYVQHEG*, YVQHEGLPK* |
| LLVCSVNGF | II | 0.987 | 1.148 | 1.334 | 1.069 | 0.053 |  |  |
| LNHHNLLVC | II | 1.272 | 1.520 | 1.816 | 1.093 | 0.035 |  |  |
| LQRTDPPRT | I | 1.025 | 1.189 | 1.379 | 1.003 | 0.972 |  | RTHMTHHAV* |
| LQWMYGCDV | I | 1.072 | 1.159 | 1.254 | 1.037 | 0.012 |  |  |
| LRGHDQYAY | I | 1.042 | 1.153 | 1.276 | 1.006 | 0.031 |  |  |
| LRGHKQYAY | I | 3.197 | 12.844 | 51.603 | 6.225 | 0.973 |  | LLRGHKQYA |
| LRGYHQFAY | I | 0.992 | 3.092 | 9.636 | 1.060 | 0.978 |  |  |
| LRGYNQLAY | I | 1.031 | 1.128 | 1.234 | 1.001 | 0.996 |  |  |
| LRGYNRLAY | I | 0.837 | 5.969 | 42.560 | 1.001 | 0.115 |  | GRLLRGYNR*, LLRGYNRLA*, RLLRGYNRL*, YNRLAYDGK*, YTCYVQHEG*, YVQHEGLPK* |
| LRNLRGYYN | I | 1.010 | 1.078 | 1.152 | 1.002 | 0.295 |  |  |
| LRRHLENGK | I | 1.047 | 1.108 | 1.171 | 1.041 | 0.944 |  |  |
| LRRYLENGK | I | 1.056 | 1.140 | 1.231 | 1.062 | 0.976 |  |  |
| LRRYLENRK | I | 0.527 | 0.704 | 0.940 | 0.935 | 0.993 |  | YLENRKKTL |
| LRTTLQRRV | II | 1.106 | 1.164 | 1.225 | 1.015 | 0.009 |  | FYPAQIKVR, YQLELRTTL, LGLIIHHRS, IIHHRSQKG, AQIKVRWFR*, VTDFYPAQI, MLSGIGGFV, LIIHHRSQK, LSGIGGFVL, GLGLIIHHR |
| LRWEPSSQS | I | 0.970 | 1.152 | 1.369 | 1.006 | 0.918 |  |  |
| LRWKPSSQP | I | 0.956 | 1.471 | 2.264 | 1.061 | 0 |  | PEPLTLRWK*, GSHTLQRMF |
| LTLRWEPSS | I | 0.476 | 0.745 | 1.165 | 0.986 | 0.996 |  |  |
| LTVEWSARS | II | 1.033 | 1.093 | 1.157 | 1.057 | 0.967 |  | FLEDRRALV |
| LVSRSIYNR | II | 1.073 | 1.115 | 1.159 | 1.052 | 0.967 |  | RVRLVSRSI*, ILERKRAAV, LLGLPAAEY, FRAVTLLGL, VRLVSRSIY*, EFRAVTLLG, IVRFDSDVG, RVCRHNYQL |
| MAAQITKRK | I | 1.022 | 1.067 | 1.114 | 1.024 | 0.881 |  |  |
| MKASAQTYR | I | 1.048 | 1.106 | 1.168 | 1.065 | 0.022 |  |  |
| NYEVAFRGI | II | 1.047 | 1.122 | 1.202 | 1.014 | 0.012 |  |  |
| PEPLTLRWK | I | 0.956 | 1.471 | 2.264 | 1.003 | 0.975 |  | LRWKPSSQP*, GSHTLQRMF |
| PGRGEPRFI | I | 0.968 | 10.698 | 118.260 | 1.294 | 0.004 |  |  |
| PIVGIVAGL | I | 0.914 | 1.315 | 1.893 | 1.011 | 0.976 |  |  |
| PKVTVYPSK | II | 0.452 | 0.700 | 1.083 | 0.968 | 0.922 |  |  |
| PRFISVGYV | I | 0.919 | 0.970 | 1.025 | 0.990 | 0.010 |  |  |
| QITQRKLEA | I | 1.012 | 1.104 | 1.203 | 1.014 | 0.939 |  | TQRKLEAAR |
| QIYKAQAQT | I | 1.071 | 1.142 | 1.217 | 1.062 | 0.432 |  | YKAQAQTDR |
| QIYKTNTQT | I | 0.842 | 1.785 | 3.783 | 1.009 | 0.154 |  |  |
| QRKLEAARE | I | 1.274 | 1.555 | 1.899 | 1.121 | 0.005 |  | RKLEAAREA, TIPNLGIVS*, PAVLAVLAV, VEWLRGYLE |
| QRKWEAARV | I | 0.871 | 0.938 | 1.010 | 0.995 | 0.022 |  |  |
| QRKWEAARW | I | 1.176 | 1.416 | 1.705 | 1.093 | 0.974 |  |  |
| QRMESRAPW | I | 2.386e-06 | 2.818e-06 | 3.329e-06 | 0.657 | 0.932 |  |  |
| QTYRENLRI | I | 1.027 | 1.284 | 1.606 | 1.078 | 0.027 |  |  |
| QYAYDGKDY | I | 0.395 | 0.608 | 0.935 | 0.962 | 0.965 |  |  |
| RAYLEDLCV | I | 1.977 | 3.682 | 6.858 | 2.082 | 0.018 |  |  |
| RAYLEGLCV | I | 0.854 | 0.904 | 0.957 | 0.952 | 0.023 |  |  |
| RFIAVGYVD | I | 0.054 | 0.423 | 3.308 | 0.810 | 0.123 |  |  |
| RFLDRHFYN | II | 1.326 | 1.685 | 2.141 | 1.302 | 0.981 |  |  |
| RFLERYFHN | II | 1.060 | 1.353 | 1.727 | 1.040 | 0.959 |  |  |
| RKLEAARAA | I | 1.029 | 1.076 | 1.126 | 1.035 | 0.004 |  | ARAAEQLRA |
| RKWEAVHAA | I | 1.058 | 1.179 | 1.313 | 1.016 | 0.014 |  |  |
| RLLRGYNRL | I | 0.837 | 5.969 | 42.560 | 1.012 | 0.008 |  | GRLLRGYNR*, LLRGYNRLA*, LRGYNRLAY*, YNRLAYDGK*, YTCYVQHEG*, YVQHEGLPK* |
| RPRFLEYST | II | 1.000 | 1.448 | 2.096 | 1.173 | 0.008 |  | FRNQKGHSG, SGLQPRGFL |
| RTHMTHHAV | I | 1.021 | 1.189 | 1.384 | 1.008 | 0.101 |  | LQRTDPPRT* |
| RVAEQLRAY | I | 0.271 | 2.998 | 33.152 | 1.510 | 0.984 |  |  |
| RVRGVTRHI | II | 1.038 | 1.087 | 1.138 | 1.034 | 0.961 |  | VLEGARASV*, YEVAYRGIL, VYQFKGLCY, IIRQRSRKG, VRGVTRHIY, LIIRQRSRK, ALNHHNLLI, LCYFTNGTE |
| RVRLLERRV | II | 1.091 | 1.211 | 1.346 | 1.015 | 0.059 |  | LVMLETVPQ, VRLLERRVH, LLERRRAAV*, VMLETVPQS* |
| RVRLVSRSI | II | 1.072 | 1.113 | 1.156 | 1.000 | 0.040 |  | LVSRSIYNR*, LLGLPAAEY, ILERKRAAV, EFRAVTLLG, VRLVSRSIY*, FRAVTLLGL, IVRFDSDVG |
| RVRYLHRGI | II | 1.023 | 1.129 | 1.246 | 1.005 | 0.078 |  | VRYLHRGIY*, YLHRGIYNQ, FLERRRAEV |
| RVYLEGRCV | I | 1.068 | 1.120 | 1.175 | 1.032 | 0.998 |  | VHAAEQRRV* |
| RVYLEGTCV | I | 1.041 | 1.272 | 1.554 | 1.023 | 0.301 |  | VYLEGTCVE* |
| RYFHTAMSR | I | 1.031 | 1.180 | 1.350 | 1.040 | 0.992 |  |  |
| RYFYTSMSR | I | 1.045 | 1.148 | 1.262 | 1.032 | 0.989 |  | YTSMSRPGR*, MRYFYTSMS, FYTSMSRPG |
| SHIIQRMYG | I | 0.993 | 1.041 | 1.092 | 1.015 | 0.991 |  |  |
| SHILQRMYG | I | 1.033 | 1.199 | 1.391 | 1.001 | 0.503 |  |  |
| SHTIQIMHG | I | 1.646 | 4.394 | 11.731 | 2.122 | 0.050 |  |  |
| SHTVQRMCG | I | 0.796 | 5.706 | 40.909 | 1.796 | 0.007 |  |  |
| SIEVRWFLN | II | 0.849 | 0.941 | 1.044 | 0.959 | 0.978 |  | FYNQEESVR |
| SIYNREEIV | II | 1.107 | 1.202 | 1.306 | 1.101 | 0.008 |  |  |
| SMRCFSTSV | I | 1.017e-05 | 1.179e-05 | 1.367e-05 | 0.845 | 0.990 |  |  |
| SQRMEPRAP | I | 0.978 | 1.729 | 3.058 | 1.004 | 0.520 |  |  |
| SSQSTVPIV | I | 1.082 | 1.275 | 1.502 | 1.046 | 0.975 |  |  |
| TAADMAAQI | I | 0.983 | 1.055 | 1.132 | 1.031 | 0.974 |  |  |
| TCVEWLRRH | I | 1.061 | 1.140 | 1.226 | 1.022 | 0.014 |  |  |
| TDFYPSHIK | II | 0.623 | 0.805 | 1.040 | 0.993 | 0.006 |  |  |
| TDRVGLRNL | I | 2.523e-06 | 2.978e-06 | 3.515e-06 | 0.829 | 0.987 |  | ECVEWLRRH |
| TGVVSTPLI | II | 1.025 | 1.097 | 1.173 | 1.050 | 0.007 |  |  |
| TIPNLGIVS | I | 1.213 | 1.441 | 1.714 | 1.042 | 0.985 |  | QRKLEAARE*, RKLEAAREA, PAVLAVLAV |
| TIQMMYGCH | I | 0.330 | 0.558 | 0.944 | 0.877 | 0.917 |  |  |
| TQTYRENLR | I | 1.028 | 1.125 | 1.231 | 1.002 | 0.984 |  |  |
| TVHIVGIIA | I | 1.053 | 1.175 | 1.311 | 1.027 | 0.932 |  |  |
| TYRESLRTA | I | 8.554e-06 | 9.939e-06 | 1.155e-05 | 0.993 | 0.012 |  |  |
| VAAVMWRRK | I | 1.037 | 1.104 | 1.175 | 1.020 | 0.981 |  |  |
| VAEQLRAYL | I | 1.005 | 1.055 | 1.106 | 1.003 | 0.992 |  |  |
| VAVVAVVAA | I | 1.094 | 4.387 | 17.584 | 1.804 | 0.986 |  |  |
| VFQFKGMCY | II | 1.056 | 1.100 | 1.147 | 1.001 | 0.110 |  |  |
| VHAAEQRRV | I | 1.044 | 1.087 | 1.132 | 1.016 | 0.005 |  | RVYLEGRCV* |
| VHNQEEYAR | II | 1.086 | 1.938 | 3.460 | 1.353 | 0.962 |  |  |
| VHPEVTVYP | II | 1.109 | 1.248 | 1.406 | 1.081 | 0.032 |  | CQVEHPSVM |
| VLEGARASV | II | 1.028 | 1.076 | 1.127 | 1.010 | 0.110 |  | ALNHHNLLI, RVRGVTRHI*, CRHNYEVAY, VRGVTRHIY, LIIRQRSRK |
| VMAVVMCRR | I | 1.034 | 1.068 | 1.104 | 1.020 | 0.978 |  |  |
| VMLETVPQS | II | 1.087 | 1.206 | 1.337 | 1.060 | 0.005 |  | LVMLETVPQ, VRLLERRVH, LLERRRAAV*, RVRLLERRV* |
| VQRRVHPKV | II | 1.096 | 1.153 | 1.212 | 1.011 | 0.011 |  |  |
| VQRRVQPKV | II | 1.064 | 1.110 | 1.158 | 1.009 | 0.991 |  | FTVQRRVQP*, RFLWQPKRE, SFTVQRRVQ |
| VRFDSDAAS | I | 1.011 | 1.110 | 1.218 | 1.026 | 0.970 |  |  |
| VRFDSDATS | I | 1.016 | 1.068 | 1.123 | 1.032 | 0.015 |  |  |
| VRFDSDVGV | II | 1.008 | 1.053 | 1.100 | 1.018 | 0.022 |  | VRGVTRHIY, LIIRQRSRK |
| VRFLDRHFY | II | 1.164 | 1.279 | 1.404 | 1.144 | 0.974 |  |  |
| VRFLDRYFY | II | 1.019 | 1.065 | 1.113 | 1.004 | 0.969 |  |  |
| VRLVSRSIY | II | 1.060 | 1.105 | 1.151 | 1.001 | 0.064 |  | ILERKRAAV, LVSRSIYNR*, RVRLVSRSI*, LLGLPAAEY |
| VRYLHRGIY | II | 1.031 | 1.130 | 1.239 | 1.012 | 0.968 |  | RVRYLHRGI*, YLHRGIYNQ |
| VTELGRPVA | II | 1.081 | 1.172 | 1.271 | 1.022 | 0.774 |  |  |
| VTHHPVSDH | I | 1.046 | 2.243 | 4.810 | 1.237 | 0.038 |  |  |
| VTRYIYNRE | II | 1.043 | 1.103 | 1.166 | 1.001 | 0.073 | ◊ |  |
| VVAAVMCRR | I | 1.051 | 1.093 | 1.137 | 1.021 | 0.883 |  | VIGAVVAAV |
| VVESFTVQR | II | 0.977 | 1.041 | 1.109 | 1.009 | 0.957 |  |  |
| VVMCRRKSS | I | 1.043 | 1.106 | 1.173 | 1.025 | 0.933 |  |  |
| VYLEGTCVE | I | 1.025 | 1.240 | 1.501 | 1.015 | 0.978 |  | RVYLEGTCV* |
| VYPAKTQPL | II | 1.044 | 1.086 | 1.130 | 1.014 | 0.965 |  |  |
| VYPSKTQPL | II | 1.131 | 1.216 | 1.308 | 1.033 | 0.941 |  |  |
| VYRAVTPLG | II | 1.031 | 1.073 | 1.117 | 1.007 | 0.048 |  | TVCRHNYQL |
| WAEQLRAYL | I | 1.068 | 1.156 | 1.251 | 1.069 | 0.989 |  |  |
| WDQETRNMK | I | 1.145 | 1.352 | 1.596 | 1.109 | 0.980 |  |  |
| WEAAHVAEQ | I | 1.038 | 1.087 | 1.139 | 1.004 | 0.037 | ◊ |  |
| WEAAREAEQ | I | 0.958 | 2.058 | 4.424 | 1.181 | 0.065 |  | TQRKWEAAR |
| WEAARRAEQ | I | 1.036 | 1.149 | 1.274 | 1.041 | 0.014 |  | RAEQLRAYL, QRKWEAARR, ARRAEQLRA* |
| WEPSSQSTV | I | 1.060 | 1.150 | 1.248 | 1.035 | 0.020 |  |  |
| WQRMYGCDL | I | 1.037 | 1.120 | 1.210 | 1.026 | 0.009 |  |  |
| YCRHNYGVG | II | 0.962 | 1.101 | 1.259 | 1.013 | 0.971 |  | CRHNYGVGE |
| YCRHNYGVV | II | 1.056 | 1.105 | 1.157 | 1.010 | 0.011 |  | YGVVESFTV |
| YEVGYRGIL | II | 1.001 | 1.073 | 1.149 | 1.003 | 0.006 |  | FYPGQIKVQ, QIKVQWFRN |
| YFDTAVSRP | I | 1.012 | 1.062 | 1.114 | 1.001 | 0.993 |  |  |
| YFFTSVSRP | I | 1.034 | 1.078 | 1.124 | 1.017 | 0.948 |  | FFTSVSRPG* |
| YFHTAMSRP | I | 1.035 | 1.080 | 1.126 | 1.017 | 0.039 |  | MRYFHTAMS, FHTAMSRPG |
| YFYNQEEYV | II | 1.018 | 1.064 | 1.111 | 1.000 | 0.008 |  | FYNQEEYVR |
| YFYTAVSRP | I | 1.031 | 1.070 | 1.110 | 1.000 | 0.002 |  | MRYFYTAVS, FYTAVSRPG* |
| YIALKEDLR | I | 1.052 | 1.092 | 1.133 | 1.042 | 0.021 |  |  |
| YIYNREEYA | II | 1.060 | 1.106 | 1.153 | 1.023 | 0.982 |  |  |
| YKRQAQTDR | I | 1.044 | 1.095 | 1.148 | 1.009 | 0.742 |  |  |
| YNQEESVRF | II | 1.042 | 1.086 | 1.131 | 1.016 | 0.037 |  |  |
| YNRLAYDGK | I | 0.837 | 5.969 | 42.560 | 1.030 | 0.003 |  | GRLLRGYNR*, LLRGYNRLA*, LRGYNRLAY*, RLLRGYNRL*, YTCYVQHEG*, YVQHEGLPK* |
| YQFKAMCYF | II | 1.187 | 1.421 | 1.700 | 1.192 | 0.986 | ◊ |  |
| YRAVTPQGR | II | 1.066 | 1.108 | 1.152 | 1.016 | 0.014 |  | LGLIIRQRS |
| YRESLRNLR | I | 1.005 | 1.041 | 1.078 | 1.027 | 0.987 | ◊ |  |
| YSTSECHFF | II | 1.093 | 1.175 | 1.264 | 1.050 | 0.984 |  |  |
| YTAVSRPGR | I | 1.055 | 1.102 | 1.150 | 1.047 | 0.126 |  |  |
| YTCYVQHEG | I | 0.837 | 5.969 | 42.560 | 1.001 | 0.082 |  | GRLLRGYNR*, LLRGYNRLA*, LRGYNRLAY*, RLLRGYNRL*, YNRLAYDGK*, YVQHEGLPK* |
| YTSMSRPGR | I | 1.045 | 1.148 | 1.260 | 1.028 | 0.016 |  | RYFYTSMSR*, MRYFYTSMS, FYTSMSRPG |
| YVQHEGLPK | I | 0.837 | 5.969 | 42.560 | 1.000 | 0.996 |  | GRLLRGYNR*, LLRGYNRLA*, LRGYNRLAY*, RLLRGYNRL*, YNRLAYDGK*, YTCYVQHEG* |

^a^Mismatches identified by Niemann M, Matern BM, Spierings E, Schaub S, Honger G. Peptides Derived From Mismatched Paternal Human Leukocyte Antigen Predicted to Be Presented by HLA-DRB1, -DRB3/4/5, -DQ, and -DP Induce Child-Specific Antibodies in Pregnant Women. Front Immunol. 2021;12:797360.

^b^Highly correlated TcEMMs (correlation > 0.74) are listed sequentially by descending degree of correlation with the Lasso selected TcEMM.

*Highly correlated TcEMM also selected by Lasso among the DCGF predictors.
