# Supplementary material for: Dissecting the impact of molecular T-cell HLA mismatches in kidney transplant failure: A retrospective cohort study
Source: Front Immunol. 2022 Nov 24;13:1067075. doi: 10.3389/fimmu.2022.1067075 (PMC9730505; doi:10.3389/fimmu.2022.1067075)
Supplement: Supplementary file 6 [file Table_5.docx]

**Supplementary Table 5. Difference in TcEMMs between the main analysis (IGMT/HLA version 3.46) and the updated PIRCHE-II algorithm (version 3.47)**

|  | TcEMM by AFT models | TcEMM by LASSO penalized regression models | | NE | Differences^a^ |
| --- | --- | --- | --- | --- | --- |
|  |  | selected | PSI |  |  |
| AAVMCRRKS |  |  |  |  | 1/8324 |
| AVMCRRKSS |  |  |  |  | 1/7278 |
| EVIVYPAKT | S |  |  | * | 1/1 |
| EVRWFRNDQ | S |  |  | * | 1/1 |
| EVRWFWNGQ | S |  |  | * | 1/1 |
| EWRAQSEPA |  | S | S | * | 3/3 |
| FCSVSGFYP |  |  |  | * | 2/2 |
| FMLGLLFLG | S |  |  | * | 1/1 |
| FQKWAAVMV |  |  |  |  | 1/6411 |
| FQNGQEEKA |  | S |  | * | 1/1 |
| FQTLVMLET |  |  |  | * | 3/3 |
| FRKGQEEKT | S |  |  | * | 1/1 |
| FRNGQEEKA |  |  |  |  | 1/996 |
| FRNGQEEKT |  |  |  |  | 8/4294 |
| FRNQKGHAG | S |  |  | * | 5/5 |
| FTVQRRVHP | S |  |  |  | 1/17038 |
| FTVQRRVQP | S | S | S |  | 2/15648 |
| FYPGQIKVQ |  |  |  |  | 1/4598 |
| FYPGQIKVR | S |  |  |  | 3/17195 |
| FYPGSIELR | S |  |  | * | 1/1 |
| GFMLGLLFL | S |  |  | * | 1/1 |
| GGFMLGLLF | S |  |  | * | 2/2 |
| GLFIYFRNQ |  | S |  | * | 1/1 |
| GMVSTGLVQ | S |  |  | * | 1/1 |
| GSIELRWFR | S |  |  | * | 1/1 |
| GSIEVRWFR |  |  |  |  | 1/597 |
| GTGLFIYFR | S |  |  | * | 3/3 |
| HAGLQPTGF | S |  |  | * | 2/2 |
| HHNLLFCSV |  |  |  | * | 2/2 |
| HHSLLVCSV | S |  |  | * | 10/10 |
| HPSVTSPLA | S |  |  | * | 1/1 |
| HSGLQPRGF |  |  |  |  | 2/1175 |
| HSGLQPTGF | S |  |  | * | 1/1 |
| HYNLLVCSV |  |  |  |  | 9/2880 |
| IGAVVAAVM | S | S |  |  | 1/6718 |
| IGAVVATVM |  |  |  |  | 1/9009 |
| IIVEWRAQS | S | S | S |  | 2/20345 |
| ITVEWRAQS |  |  |  |  | 2/1135 |
| IVGIIAGLL | S |  |  | * | 1/1 |
| IVGIIAGLV |  |  |  |  | 1/2212 |
| KMLSGIGGF |  |  |  |  | 1/1773 |
| KMLSGVGGF | S |  |  | * | 1/1 |
| KTGVVSTGL | S |  |  |  | 1/7803 |
| KVTVYPSKS | S |  |  | * | 1/1 |
| LAVEWRARS |  |  |  | * | 17/17 |
| LFCSVSGFY |  |  |  | * | 2/2 |
| LIHSGDWTF |  |  |  | * | 2/2 |
| LIQNGDWNF | S |  |  | * | 2/2 |
| LIQNGDWTF | S | S | S |  | 2/8883 |
| LLFLGTGLF | S |  |  | * | 4/4 |
| LQHHNLLFC |  |  |  | * | 2/2 |
| LQHHSLLVC | S |  |  | * | 2/2 |
| LQHYNLLVC |  |  |  |  | 1/311 |
| LRWEPSSQS |  | S |  |  | 5/590 |
| LRWFRNGQE | S |  |  | * | 1/1 |
| LSGIGGFVL |  |  |  |  | 3/8874 |
| LSGVGGFML |  |  |  | * | 6/6 |
| LTVEWRAQS |  |  |  | * | 3/3 |
| LTVEWRARS |  |  |  |  | 8/4075 |
| LTVEWRAWS | S |  |  | * | 3/3 |
| LTVEWSARS | S | S |  |  | 23/7006 |
| LVCSVTDFY | S |  |  |  | 1/5861 |
| LVMLEMTPQ |  |  |  |  | 1/242 |
| LVMLETVPW |  |  |  | * | 6/6 |
| LVMLETVSR | S |  |  | * | 1/1 |
| LVQNGDWTF | S |  |  | * | 1/1 |
| MLEMTPQCG |  |  |  | * | 4/4 |
| MLEMTPQHG |  |  |  |  | 1/6308 |
| MLEMTPQRG |  |  |  |  | 2/2252 |
| MLSGIGGFV | S |  |  |  | 3/9889 |
| MLSGVGGFM |  |  |  | * | 2/2 |
| MSPLTVEWR | S |  |  |  | 1/4525 |
| MVSTGLIQN |  |  |  |  | 1/2386 |
| NFQTLVMLE | S |  |  | * | 1/1 |
| NLLFCSVSG |  |  |  | * | 2/2 |
| NLLVCSVSG |  |  |  |  | 1/2647 |
| PLAVEWRAR | S |  |  | * | 3/3 |
| PLQHHNLLF |  |  |  | * | 2/2 |
| PLQHHNLLV | S |  |  | * | 1/1 |
| PLQHHSLLV | S |  |  | * | 11/11 |
| PLQHYNLLV |  |  |  |  | 11/3169 |
| PRVTVYPSK | S |  |  | * | 2/2 |
| PSLQNPIIV |  |  |  |  | 1/5560 |
| PSVTSPLAV |  |  |  | * | 3/3 |
| QIKVQWFRN |  |  |  |  | 1/3442 |
| QRRVHPEVI | S |  |  | * | 4/4 |
| QRRVQPKVT |  |  |  |  | 1/2760 |
| QSKMLSGIG |  |  |  |  | 1/156 |
| RVTVYPSKT |  |  |  | * | 3/3 |
| SEPAQSKML |  |  |  | * | 6/6 |
| SESAQSKML | S |  |  | * | 3/3 |
| SFTVQRRVQ | S |  |  |  | 1/13606 |
| SGLQPRGFL |  |  |  |  | 1/143 |
| SGLQPTGFL | S |  |  | * | 2/2 |
| SIEVRWFRK |  |  |  | * | 2/2 |
| SIEVRWFRN |  |  |  |  | 1/595 |
| SIEVRWFWN | S |  |  | * | 1/1 |
| SLLVCSVSG | S |  |  | * | 1/1 |
| SQPLQHHNL | S |  |  | * | 1/1 |
| TGLFIYFRN | S |  |  | * | 3/3 |
| TLVMLETVP |  |  |  | * | 4/4 |
| TQPLQHHSL | S |  |  | * | 5/5 |
| TQPLQHYNL |  |  |  |  | 4/1318 |
| VCSVSGFYP | S |  |  |  | 1/1126 |
| VGGFMLGLL | S |  |  | * | 1/1 |
| VIGAVVAAV | S |  |  |  | 1/12166 |
| VIVYPAKTQ | S |  |  | * | 2/2 |
| VMLEMTPQC |  |  |  | * | 3/3 |
| VMLEMTPQH |  |  |  |  | 2/22844 |
| VMLEMTPQR |  |  |  |  | 4/4738 |
| VMLETVPWS |  |  |  | * | 7/7 |
| VMLETVSRS | S |  |  | * | 1/1 |
| VQRRVQPKV | S | S |  |  | 2/14970 |
| VQRRVQPRV |  |  |  | * | 5/5 |
| VRWFQNGQE |  |  |  | * | 1/1 |
| VRWFRKGQE |  |  |  | * | 2/2 |
| VRWFRNGQE |  |  |  |  | 1/1838 |
| VRWFWNGQE | S |  |  | * | 2/2 |
| VVAAVMCRR | S | S |  |  | 4/16490 |
| VVATVMCRR |  |  |  |  | 5/17597 |
| VVSTGLIHN |  |  |  |  | 1/5297 |
| VVSTGLIHS |  |  |  | * | 2/2 |
| VVSTGLIQN |  |  |  |  | 2/7856 |
| VVSTGLIRN | S |  |  | * | 1/1 |
| VYPAKTQPL | S | S |  |  | 2/20333 |
| VYPSKSQPL | S |  |  | * | 2/2 |
| VYPSKTQPL | S | S |  |  | 5/6156 |
| VYTCHVEHP |  |  |  |  | 1/356 |
| WAAVMVPSG |  |  |  |  | 1/4071 |
| WEPSSQSTI |  |  |  |  | 1/6947 |
| WEPSSQSTV | S | S | S |  | 1/3359 |
| WFRKGQEEK | S |  |  | * | 1/1 |
| WFWNGQEEK | S |  |  | * | 1/1 |
| WNFQTLVML | S |  |  | * | 1/1 |
| WRAQSEPAQ |  |  |  | * | 9/9 |
| WRAWSESAQ | S |  |  | * | 2/2 |
| WSESAQSKM | S |  |  | * | 2/2 |
| WTFQTLVML |  |  |  |  | 1/273 |
| YFRNQKGHA | S |  |  | * | 7/7 |
| YNLLVCSVS |  |  |  |  | 9/2508 |
| YSQAAYSDS | S |  |  | * | 1/1 |
| YSQAVSSDS | S |  |  | * | 1/1 |
| YTCYVQHEG |  | S |  | * | 1/1 |
| YVQHEGLPK |  | S |  | * | 1/1 |

^a^Differences represent the ratio between the number of donor:recipient pairs with different TcEMM (between version 3.47 and 3.46) and the total number of pairs expressing the TcEMM

Abbreviation: PSI, significance in the post-selection inference test; NE, whether the cores are not observed in the updated 3.47 PIRCHE-II algorithm; S, TcEMM statistically significantly associated with DCGF in AFT models, selected by the Lasso regression models, or deemed significant by PSI.
